# Ozanimod (RPC1063), a selective S1P_R1_ and S1P_R5_ modulator, reduces chronic inflammation and alleviates kidney pathology in murine systemic lupus erythematosus

**DOI:** 10.1371/journal.pone.0193236

**Published:** 2018-04-02

**Authors:** Kristen R. Taylor Meadows, Marcos W. Steinberg, Bryan Clemons, Matthew E. Stokes, Gregory J. Opiteck, Robert Peach, Fiona L. Scott

**Affiliations:** 1 Receptos, a wholly owned subsidiary of Celgene Corporation, San Diego, California, United States of America; 2 Celgene Corporation, Seattle, Washington, United States of America; Instituto Nacional de Ciencias Medicas y Nutricion Salvador Zubiran, MEXICO

## Abstract

Ozanimod (RPC1063) is a specific and potent small molecule modulator of the sphingosine 1-phosphate receptor 1 (S1P_R1_) and receptor 5 (S1P_R5_), which has shown therapeutic benefit in clinical trials of relapsing multiple sclerosis and ulcerative colitis. Ozanimod and its active metabolite, RP-101075, exhibit a similar specificity profile at the S1P receptor family *in vitro* and pharmacodynamic profile *in vivo*. The NZBWF1 mouse model was used in therapeutic dosing mode to assess the potential benefit of ozanimod and RP-101075 in an established animal model of systemic lupus erythematosus. Compared with vehicle-treated animals, ozanimod and RP-101075 reduced proteinuria over the duration of the study and serum blood urea nitrogen at termination. Additionally, ozanimod and RP-101075 reduced kidney disease in a dose-dependent manner, as measured by histological assessment of mesangial expansion, endo- and exo-capillary proliferation, interstitial infiltrates and fibrosis, glomerular deposits, and tubular atrophy. Further exploration into gene expression changes in the kidney demonstrate that RP-101075 also significantly reduced expression of fibrotic and immune-related genes in the kidneys. Of note, RP-101075 lowered the number of plasmacytoid dendritic cells, a major source of interferon alpha in lupus patients, and reduced all B and T cell subsets in the spleen. Given the efficacy demonstrated by ozanimod and its metabolite RP-101075 in the NZBWF1 preclinical animal model, ozanimod may warrant clinical evaluation as a potential treatment for systemic lupus erythematosus.

## Introduction

Systemic lupus erythematosus (SLE) is a complex systemic autoimmune disease characterized by the production of autoantibodies and immune complex deposition in a multitude of tissues including kidney, skin, joints, heart, lungs, and brain. Both B and T cells drive chronic inflammation, and cellular trafficking of lymphocytes to tissues and organs plays a major role in SLE [1–4].

The sphingosine 1-phosphate (S1P) receptor 1 (S1P_R1_), a member of the G-protein–coupled receptors, is widely expressed on many cell types [5]. S1P_R1_ signaling plays a pivotal role in the trafficking of B and T lymphocytes [6]. Pharmacological activation of S1P_R1_ results in selective and reversible sequestration of C-C chemokine receptor type 7 (CCR7)^+^ lymphocyte subsets in peripheral lymphoid tissue [7–9]. This sequestration prevents migration of autoreactive lymphocytes to areas of inflammation [10,11]. Importantly, CCR7^-^ T cell subsets (effector memory T cells and terminally differentiated effector memory T cells) are spared, maintaining immune surveillance [10]. Targeting S1P_R1_ with small molecule drugs has proven therapeutic utility in the treatment of relapsing multiple sclerosis (MS) [12–14] and ulcerative colitis [15]. Other S1P_R1_-dependent mechanisms that could provide benefit in SLE include suppression of the interferon (IFN)-driven cytokine storm [16,17]. Indeed fingolimod (Gilenya, FTY720), a non-selective S1P receptor modulator, was efficacious in reducing proteinuria and improving kidney histology in NZBWF1, BXSB, and MRL-lpr/lpr mouse models of SLE [18–20].

Agonism of S1P_R1_ has recently been shown to modulate IFN alpha (IFNα) signaling, a critical inflammatory driver in autoimmune diseases including type 1 diabetes and SLE. Teijaro *et al*. demonstrated that S1P_R1_ modulation by CYM5443, an S1P_R1_ agonist, mediates IFNAR1 degradation and inhibits IFNα autoamplification by plasmacytoid dendritic cells (pDC) [21]. Furthermore, CYM5443 also limits the migration of autoimmune cells to pancreatic islets in a mouse model of type 1 diabetes (*Rip*-LCMV T1D model), preserving insulin production and maintaining glucose regulation [22]. Therefore, modulation of pDC, IFNα, and autoreactive lymphocytes suggest that the mechanism of action of S1P_R1_ modulators may be efficacious in SLE.

Ozanimod (RPC1063) is a specific and potent small molecule modulator of S1P_R1_ and S1P receptor 5 (S1P_R5_) that is therapeutically beneficial in clinical trials in relapsing MS [12–14] and ulcerative colitis [15]. To test the hypothesis that the physiological mechanisms of ozanimod would be effective in the NZBWF1 murine SLE model, we initiated two separate *in vivo* NZBWF1 studies to test both RPC1063 and its metabolite RP-101075. Characterization of multiple parameters in this model, including in-life proteinuria, terminal kidney histology, autoantibody titers, kidney gene expression and immunophenotyping, were analyzed to better understand the potential mechanism by which S1P_R1_ and S1P_R5_ modulation confers efficacy in SLE. Together, data utilizing both RPC1063 and its metabolite RP-101075 would support the likelihood that ozanimod may have clinical utility in patients with SLE.

## Materials and methods

### S1P receptor signaling assays

S1P receptor signaling assays were performed as previously described [10]. In brief, for GTPγS binding assays, 1–5 μg/well of membrane protein was incubated with 10 μM GDP, 100-500 μg/well Wheat Germ Agglutinin PVT SPA beads (Perkin Elmer) in 50 mM HEPES, 100 mM NaCl, 10 mM MgCl_2_, 20 μg/ml saponin, and 0.1% fatty acid free bovine serum albumin for 15 minutes in 96-well plates. After the addition of compound and 200 pM GTPγ[^35^S] (Perkin Elmer, 1250 Ci/mmol), the plates were incubated for 120 minutes and centrifuged at 300 × *g* for 5 minutes. Radioactivity was detected with a TopCount Instrument (Packard Instruments). Tango™ EDG6/S1PR4-*bla* U2OS cells were obtained from Life Sciences. S1P_R2_ GeneBLAzer^®^ CRE-*bla* CHO-K1 and S1P_R3_/Gα16 GeneBLAzer^®^ NFAT-*bla* CHO-K1 cells were described elsewhere [23]. All data were fit with a four-parameter variable slope non-linear regression (GraphPad Prism) to generate half-maximal effective concentration (EC_50_) and maximum efficacy relative to S1P.

### NZBWF1 study design

All *in vivo* studies were performed under an approved Animal Care and Use Committee according to Animal Research: Reporting of *In Vivo* Experiments (ARRIVE) guidelines [24]. Hooke Laboratories IACUC approved all NZBWF1 animal studies. Animals were housed in an Association for Assessment and Accreditation of Laboratory Animal Care (ALAAC)-accredited facility with free access to food and water, on a 12-hour light cycle. NZBWF1 female mice (n = 110) were obtained from The Jackson Laboratory, and mice were acclimated for 7 weeks. Proteinuria was assessed weekly using urine test strips and scored from 0–4 where 0 = no protein; 1 = trace protein (<30 mg/dL); 2 = 30–100 mg/dL; 3 = 100–500 mg/dL; and 4 = >500 mg/dL (Roche Diagnostics Chemstrip 2GP). Body weight measurements were obtained weekly starting at 20 weeks of age. At 23 weeks, the average proteinuria score was 0.8 and 0.74, respectively, for the ozanimod and RP-101075 NZBWF1 studies. At that time, mice were assigned to groups to achieve similar average body weight and proteinuria measurements (Table 1), and mice were dosed with either RPC1063 or RP-101075 daily via oral gavage from week 23 until week 42 (20 weeks total). Compounds were formulated in 5% DMSO (Sigma), 5% Tween20 (Fisher) and 90% H_2_O and this vehicle was used in the control animals. Some mice were not included in specific analyses or time points due to dosing complications or death (Table 2). Mice in group 6 were sacrificed at week 23 to serve as a baseline control group. Blood samples were obtained by retro-orbital bleed at weeks 23, 31, and 36.5 and by cardiac puncture at the end of study at week 42. Serum was isolated for anti-dsDNA antibody concentration by enzyme-linked immunosorbent assay (ELISA) according to the manufacturer’s instructions (Shibayagi Co, Ltd kit), and blood urea nitrogen (BUN) by hematology analyzer. At termination, kidneys were removed and weighed (Figure A in S1 File), the left kidneys used for histology, and the right kidneys for gene expression analysis. Spleens were shipped overnight for subsequent splenocyte analysis.

**Table 1:** Groups and treatment.

Group	N	Treatment	Dose	Route and frequency	Purpose
**Study using RPC1063**					
1	20	Vehicle	-	PO QD	Negative control
2	20	Cyclophosphamide	50 mg/kg^a^	IP QW	Positive control
3	20	RPC1063	0.3 mg/kg	PO QD	Test
4	20	RPC1063	1.0 mg/kg	PO QD	Test
5	20	RPC1063	3.0 mg/kg	PO QD	Test
6	10	None (Baseline)	-	-	Tissue source^b^
**Study using RP-101075**					
1	20	Vehicle	-	PO QD	Negative control
2	20	Cyclophosphamide	50 mg/kg^a^	IP QW	Positive control
3	20	RP-101075	0.3 mg/kg	PO QD	Test
4	20	RP-101075	1.0 mg/kg	PO QD	Test
5	20	RP-101075	3.0 mg/kg	PO QD	Test
6	10	None (Baseline)	-	-	Tissue source^b^

IP, intraperitoneal; PO, oral; QD, daily; QW, weekly.

^a^ Cyclophosphamide treatment was introduced gradually to reduce toxicity. Doses were 10 mg/kg on week 23, 20 mg/kg on week 24, then 50 mg/kg thereafter.

^b^ Group 6 animals were sacrificed at week 23.

**Table 2:** Animal deaths.

Group	Treatment	N at week 23^a^	N at week 42^b^	Lost during study	Reason for death	
					Dosing complication/ unknown^c^	Severe disease, euthanasia
**Study using RPC1063**						
1	Vehicle	20	13	8	0	8
2	Cyclophosphamide	20	20	0	0	0
3	0.3 mg/kg RPC1063	20	18	2	0	2
4	1.0 mg/kg RPC1063	20	18	2	0	2
5	3.0 mg/kg RPC1063	19	18	2	0	2
**Study using RP-101075**						
1	Vehicle	20	17	3	0	3
2	Cyclophosphamide	20	19	1	0	1
3	0.3 mg/kg RP-101075	20	18	2	0	2
4	1.0 mg/kg RP-101075	20	16	4	3	1
5	3.0 mg/kg RP-101075	19	18	1	0	1

^a^ Treatment start at week 23.

^b^ Terminal sacrifice at week 42.

^c^ Deaths for unknown reasons were not associated with high clinical scores.

### Peripheral lymphocyte counts

Blood samples were analyzed for circulating lymphocyte reduction by flow cytometry. Blood was collected into tubes containing EDTA and stained for B and T cell populations with the following antibodies: CD4 APC, CD8 PE, CD19 FITC (BD Biosciences). Following staining, red blood cells were lysed and cells were re-suspended in phosphate-buffered saline. CountBright absolute counting beads (Life Technologies) were added to each sample and analyzed on a FACSCalibur flow cytometer (BD Biosciences).

### Histology

The left kidneys were isolated, weighed, fixed in 4% paraformaldehyde and stained with hematoxylin and eosin and periodic acid-Schiff. The kidneys were evaluated and scored for multiple morphological features of lupus nephritis (mesangial expansion, endocapillary proliferation, glomerular deposits, extracapillary proliferation, interstitial infiltrates, tubular atrophy, interstitial fibrosis) by a blinded pathologist [18].

Each of the seven lesions was scored using a 4-point scale: 0 = not present, 1 = mild, 2 = moderate, and 3 = severe, with an average score calculated per animal per group. For immunoglobulin (IgG) immunohistochemistry (IHC), formalin-fixed paraffin-embedded slides were incubated at 60°C for 1 hour, rehydrated (serially in xylenes, alcohols, and water), and washed for 4 minutes in tris-buffered saline (TBS). After incubation in a steamer with citrate buffer at pH 6.0, the slides were rinsed three times with distilled water, then three times with TBS for 4 minutes each, endogenous peroxidase blocked with 3% H_2_O_2_ in methanol for 15 minutes, and the rinsing procedure repeated. A protein block was applied for 30 minutes, followed by treatment for 30 minutes with Envision+ System HRP Labelled Polymer Anti-Mouse IgG RTU (Dako). The slides were washed once with TBS containing 0.01% Tween, then twice with TBS for 4 minutes each before staining with 3,3'diaminobenzidine (DAB), then rinsed with distilled water to stop the DAB reaction. After counterstaining the nuclei in Mayer’s hematoxylin and blue in 0.1% lithium carbonate, the slides were dehydrated, cleared, and mounted.

For complement C3 antibody IHC, frozen mouse kidney samples were post fixed in pre-chilled acetone at -20°C for 10 minutes, then brought to room temperature over 1 hour and washed with TBS for 4 minutes. The samples were quickly rinsed three times with distilled water, then three times with TBS for 4 minutes each, endogenous peroxidase blocked with 3% H_2_O_2_ in methanol for 15 minutes, and the rinsing procedure repeated. A protein block was applied for 30 minutes, followed by treatment with either C3 antibody as the primary (Abcam) or rabbit IgG as the isotype control (each at 1.33 μg/mL) at 4°C overnight. The samples were washed once with TBS containing 0.01% Tween, then twice with TBS for 4 minutes each, followed by treatment for 30 minutes with Envision+ System HRP Labelled Polymer Anti-Rabbit RTU (Dako). The slides were washed once with TBS containing 0.01% Tween, then twice with TBS for 4 minutes each before staining with DAB, then rinsed with distilled water to stop the DAB reaction. After counterstaining the nuclei in Mayer’s hematoxylin and blue in 0.1% lithium carbonate, the slides were dehydrated, cleared, and mounted.

### Concentration of anti-dsDNA antibodies in serum

ELISA for anti-dsDNA antibodies was performed using an anti-dsDNA ELISA kit (Shibayagi Co., Ltd.) following manufacturer’s protocol. Results were expressed as optical density and as arbitrary units per mL. The ELISA standard curve was generated from a pool of serum from several NZBWF1 mice from a previous study run with a known high concentration of anti-dsDNA antibodies. The highest concentration used in the standard curve was 1:100 of that serum. All test samples were diluted 1000-fold for testing. The analysis of data was done with http://www.elisaanalysis.com.

### Gene expression

The right kidneys (5–9 animals per group) were frozen in RNAlater (Ambion) and stored at -80°C. Samples were shipped to Affymetrix for tissue processing and mRNA analysis of inflammation, fibrosis, and IFN-related genes using the QuantiGene Plex assay platform. Raw gene expression values were normalized to the geometric mean of housekeeping genes *PPIB*, *RPL19*, and *GAPDH*. Unsupervised clustering of gene expression profiles was performed using the *heatmap*.*plus* package in R.

### Splenocyte analysis

Sterile splenocyte suspensions were prepared by mechanically disassociating spleens in RPMI1640 + 2% fetal calf serum. Red blood cells were lysed and cell suspensions were reconstituted in complete culture media (RPMI1640, 10% fetal calf serum, L-glutamine, penicillin/streptomycin, beta-mercaptoethanol, non-essential amino acids, and sodium pyruvate) and shipped overnight on cold packs. Upon receipt, live cells were counted using a Cellometer Auto 2000 cell imager (Nexcelom Bioscience), and stained for immune cell populations including B cells, T cells, and pDCs using an Attune NxT Flow Cytometer (ThermoFisher). The following antibodies were purchased from Biolegend: B220 (clone RA3-6B2), PDCA1 (clone 927), subunit 1 of the IFNα/β receptor (IFNAR1) (clone MAR1-5A3), CD3 (clone 17A2), CD4 (clone RM2-5), CD44 (clone IM7), CD62L (clone MEL-14), CD8 (clone 53-6.7), CD19 (clone 6D5), IgM (clone RMM-1), GL7 (clone GL7), CD23 (clone B3B4), CD138 (clone 281–2), CD21 (clone 7E9), IgD (clone 11-26c.2a). The following antibodies were purchased from eBioscience: Siglec H (clone eBio440c), and CD11c (clone N418). Analysis of the splenocytes was performed using FlowJo vX software (FlowJo, LLC). A live gate followed by a doublet exclusion gate was performed for all samples. For B cell subsets, events were first gated on CD3^-^, CD19^+^. Marginal Zone B cells were IgM^+^, CD21^+^. Germinal Center B cells were B220^+^, GL-7^+^. Follicular B cells were IgM^-^, CD21^-^. Plasma cells were CD19^lo^, CD138^+^, B220^-^, and IgD^-^. All T cells were CD3^+^. Activated CD4 T cells were CD4^+^, CD44^+^, and CD62L. Naïve CD4 T cells were CD4^+^, CD44^-^, and CD62L^+^. pDCs were gated on SiglecH^+^, CD11c^+^, B220^+^, and PDCA1^+^. pDCs were also extracellularly stained with IFNAR1.

### Statistical analysis

GraphPad Prism version 7.01 for Windows was used for all data analysis and graphical illustrations. Comparisons between groups were made using one-way analysis of variance (ANOVA) with Dunnett’s comparison unless otherwise indicated. Statistical probability (*p*) values of <0.05 were considered significant.

## Results

### Improved renal function with RPC1063 and RP-101075 treatment

**Fig 1:**
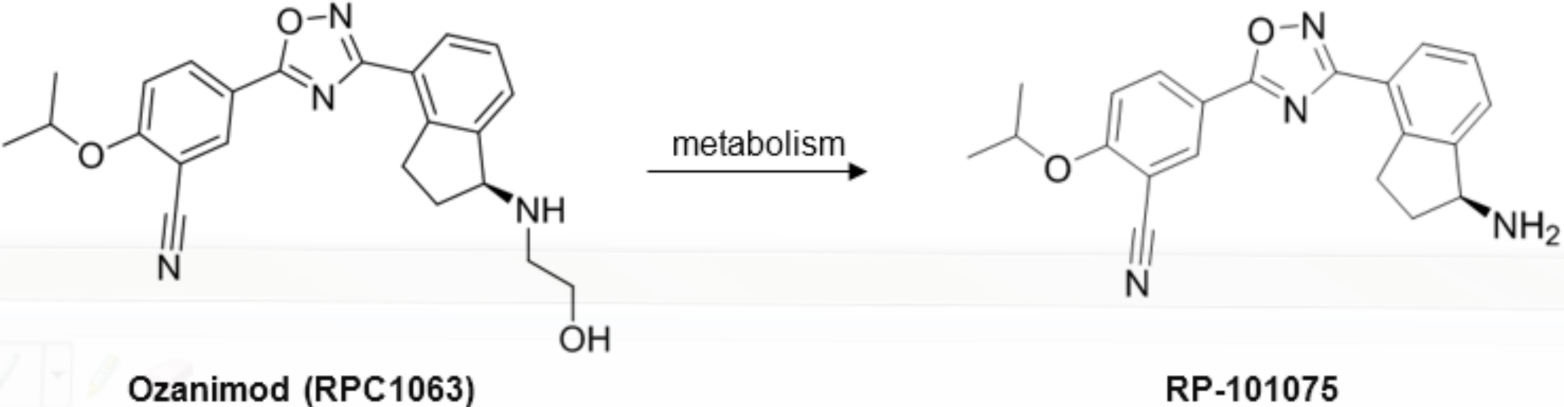
Chemical structure of ozanimod (RPC1063) and RP-101075.

**Fig 2:**
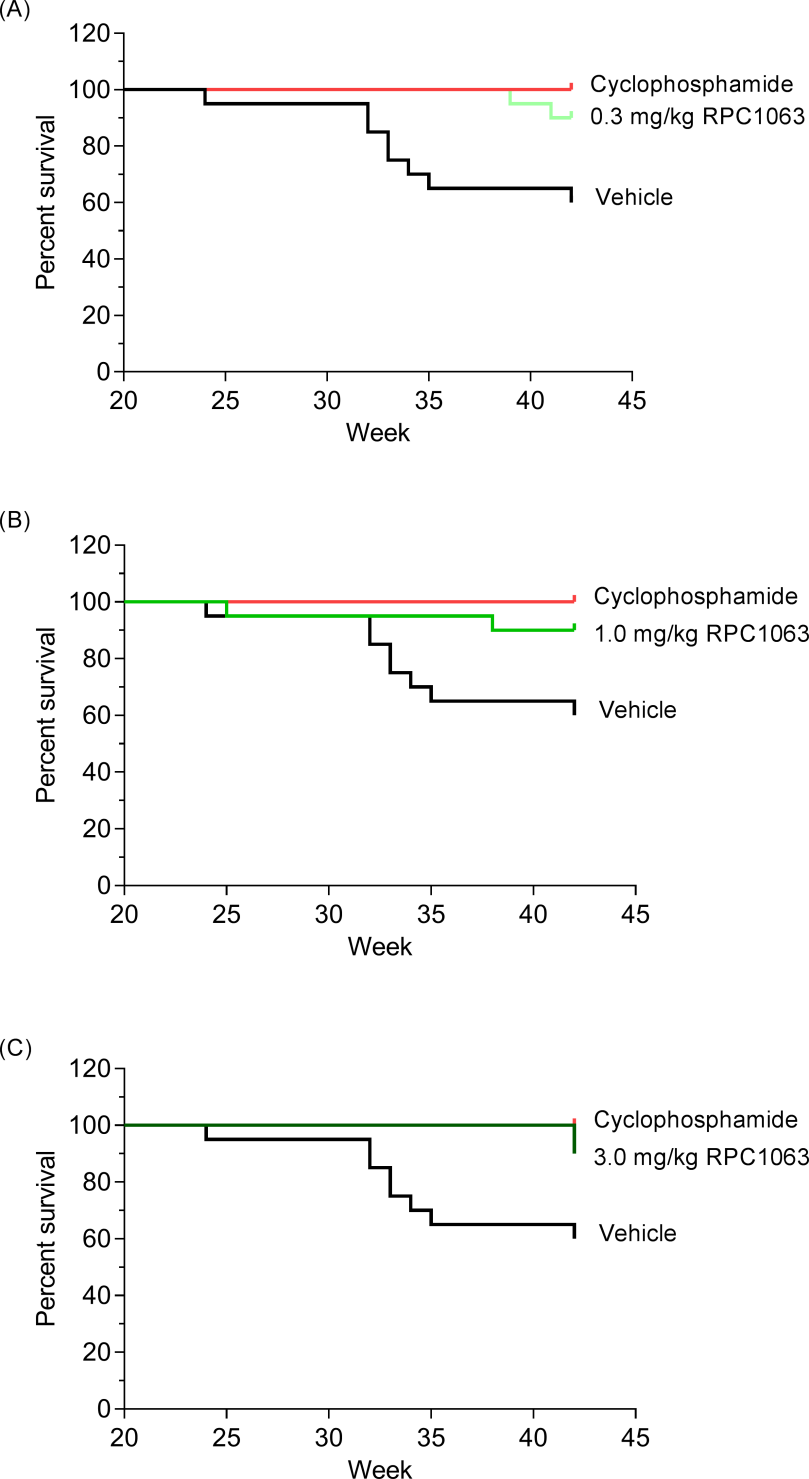
Percent survival of NZBWF1 mice over the 42-Week study. Treatment was started for all groups at week 23. Mice treated with vehicle (black line), cyclophosphamide (red line), and (A) 0.3 mg/kg RPC1063, (B) 1.0 mg/kg RPC1063, and (C) 3.0 mg/kg RPC1063 (green lines).

**Fig 3:**
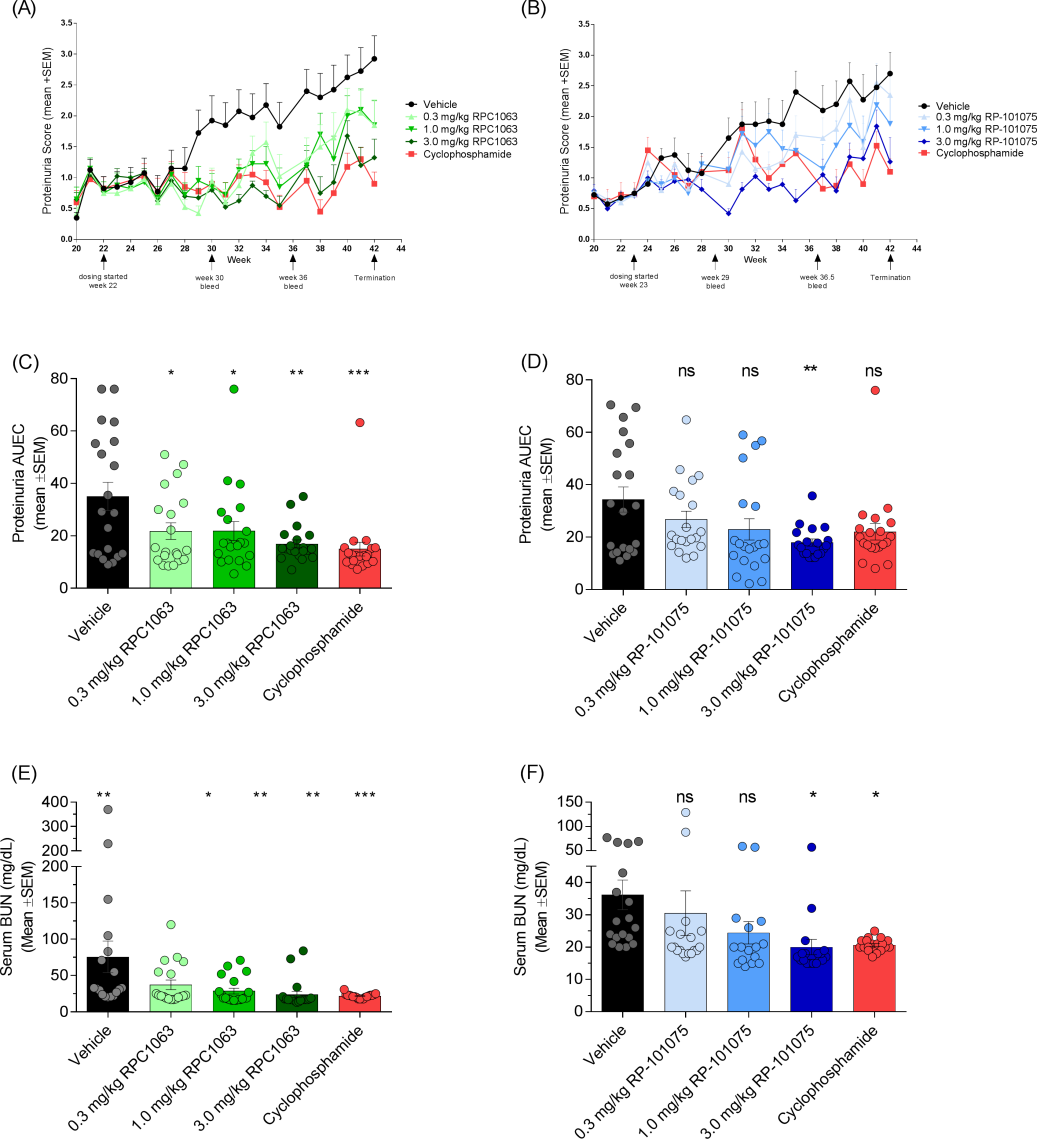
Effect of treatment on kidney function. Weekly proteinuria scores for (A) RPC1063 and (B) RP-101075 treated mice. Scores for animals that died mid-study were given a maximum score of 4.0 for the remainder of the study. Cumulative proteinuria area under the effect curve (AUEC) for (C) RPC1063, and (D) RP-101075. Serum blood urea nitrogen (BUN) at termination for (E) RPC1063 and (F) RP-101075. The normal range for mouse serum BUN levels is approximately 8–33 mg/dL (https://www.ahc.umn.edu/rar/refvalues.html). Data presented as mean ± SEM. *p <0.05, **p <0.01, ***p <0.001, ****p <0.0001, ns = not significant, for treatment versus vehicle-treated animals.

RP-101075 is a metabolite of ozanimod (RPC1063) (Fig 1) and demonstrates similar receptor specificity as RPC1063 (Table 3). Mice treated with RPC1063 demonstrate reduced peripheral circulating lymphocytes [10] and RP-101075 results in a similar reduction in peripheral circulating blood lymphocytes (Figure B in S1 File), consistent with the predicted *in vivo* mechanism of action of S1P_R1_ modulators. In addition, cyclophosphamide reduced circulating B cells (Figure B in S1 File), consistent with its cytotoxic effect on circulating B cells [25]. Animals treated with RPC1063 over the 20 week study demonstrated significant improvement in survival (Fig 2, Table 2). RP-101075 demonstrated a trend in improved survival with dosing, however it was not significant (Table 2). Compared with vehicle-treated animals, mice treated with RPC1063 and RP-101075 showed a dose-dependent reduction in weekly proteinuria scores, with all doses of RPC1063 and 3.0 mg/kg RP-101075 demonstrating a significant reduction in cumulative area under the effect curve (Fig 3A–3D). Furthermore, RPC1063 and RP-101075 significantly reduced serum BUN at termination (Fig 3E and 3F). Overall, the improvement in proteinuria and serum BUN with RPC1063 and RP-101075 treatment was equivalent to animals receiving the positive control, cyclophosphamide. In addition, the improvement in kidney health correlated with reduced mortality due to progressive and chronic kidney damage in the treatment groups (Table 2).

**Table 3:** Specificity of ozanimod and its metabolite RP-101075 on S1P receptors.

EC_50_, nM (Mean ±SD)	S1P_R1_^a^	S1P_R2_^b^	S1P_R3_^c^	S1P_R4_^d^	S1P_R5_^a^
Ozanimod (RPC1063)	0.41 ±0.16	>10,000	>10,000	>7,865	11 ±4.3
RP-101075	0.27 ±0.06	>7,720	>10,000	>10,000	5.9 ±1.0

EC_50_, 50% effective concentration; SD, standard deviation.

Data represent n = 2–7 independent experiments for all receptors in all signaling pathways.

^a^GTPγS

^b^cAMP signaling

^c^Calcium signaling

^d^β-arrestin.

### RPC1063 and RP-101075 reduce inflammation and improve kidney morphology

**Fig 4:**
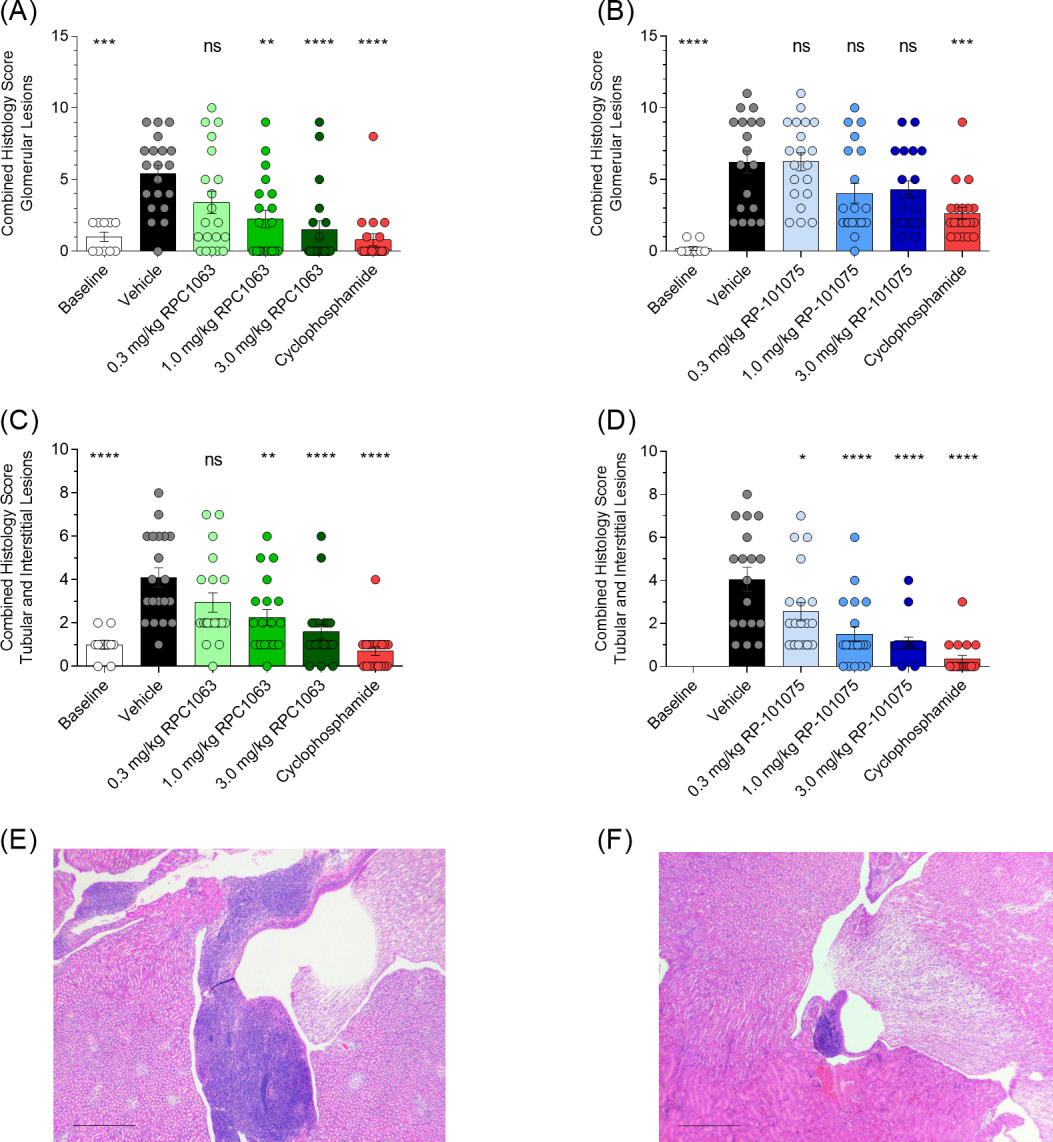
Effect of treatment on histological changes in the kidney measured at termination. Combined histology score for glomerular lesions (includes mesangial expansion, endocapillary proliferation, glomerular deposits and extracapillary proliferation) for (A) RPC1063 and (B) RP 101075. Combined histology score for tubular and interstitial lesions (includes interstitial infiltrates, tubular atrophy and interstitial fibrosis) for (C) RPC1063 and (D) RP-101075. (E) Inflammatory infiltrates in a representative (E) vehicle-treated animal and (F) RPC1063 3 mg/kg-treated animal. Calibration bars (E,F) 1 mm, 4× objective, 11×s1p magnification. Histology data presented as mean ± SEM. Baseline data collected from NZBWF1 mice sacrificed at week 23 prior to dosing initiation. *p <0.05, **p <0.01, ***p <0.001, ****p <0.0001, ns = not significant, for treatment versus vehicle-treated animals.

Kidneys were isolated and fixed for histological analysis at termination. RPC1063 and RP-101075 reduced several features of histological changes within the kidney, including mesangial expansion, glomerular deposits, interstitial infiltrates, tubular atrophy, and interstitial fibrosis, showing statistically significant improvements compared with vehicle-treated animals (Tables 4 and 5, Fig 4). Across the individual features of histological changes and overall (combined score), the decrease in severity of lesions was dose-related for RPC1063 (Table 4) and RP-101075 (Table 5).

**Table 4:** Histology scoring: RPC1063.

Treatment	Morphological lupus nephritis feature (mean ±SEM)							
	Mesangial expansion	Endocapillary proliferation	Glomerular deposits	Extracapillary proliferation	Interstitial infiltrates	Tubular atrophy	Interstitial fibrosis	Combined score
Baseline	0 ±0****	0.5 ±0.2 ****	0.5±0.2 ***	0 ±0 ^ns^	1.0 ±0.2 ***	0 ±0 **	0 ±0 **	2.0 ±0.5 ****
Vehicle	0.8 ±0.1	1.9 ±0.2	2.0 ±0.2	0.8 ±0.2	2.2 ±0.2	1.4 ±0.3	0.6 ±0.2	9.5 ±1.0
0.3 mg/kg RPC1063	0.5 ±0.1 ^ns^	1.2 ±0.2 *	1.1 ±0.3 *	0.7 ±0.2 ^ns^	1.9 ±0.2 ^ns^	0.8 ±0.2 ^ns^	0.3 ±0.1 ^ns^	6.4 ±1.2 ^ns^
1.0 mg/kg RPC1063	0.3 ±0.1 **	0.9 ±0.2 ***	0.8 ±0.2 **	0.3 ±0.2 ^ns^	1.5 ±0.2 **	0.7 ±0.2 ^ns^	0.2 ±0.1 *	4.5 ±1.0 ***
3.0 mg/kg RPC1063	0.2 ±0.1 ***	0.5 ±0.2 ****	0.6 ±0.2 ****	0.3 ±0.2 ^ns^	1.2 ±0.2 ****	0.4 ±0.2 **	0.1 ±0.1 **	3.1 ±0.9 ****
Cyclophosphamide	0.1 ±0.1 ****	0.2 ±0.1 ****	0.4 ±0.2 ****	0.2 ±0.2 ^ns^	0.6 ±0.1 ****	0.1 ±0.1 ***	0.1 ±0.1 **	1.5 ±0.6 ****

SEM, standard error of the mean.

Note: scores for animals that died on-study were censored.

*p <0.05

**p <0.01

***p <0.001

****p <0.0001

ns = not significant, for treatment versus vehicle-treated animals.

**Table 5:** Histology scoring: RP-101075.

Treatment	Morphological lupus nephritis feature (mean ±SEM)							
	Mesangial expansion	Endocapillary proliferation	Glomerular deposits	Extracapillary proliferation	Interstitial infiltrates	Tubular atrophy	Interstitial fibrosis	Combined score
Baseline	0 ±0 ***	0.1 ±0.1 ****	0.1 ±0.1 ****	0 ±0 *	not scored	not scored	not scored	NA
Vehicle	1.3 ±0.2	2.2 ±0.2	2.0 ±0.2	0.7 ±0.2	2.4 ±0.2	1.1 ±0.3	0.4 ±0.1	10.3 ±1.3
0.3 mg/kg RP-101075	1.3 ±0.2 ^ns^	2.4 ±0.2 ^ns^	2.1 ±0.2 ^ns^	0.5 ±0.2 ^ns^	1.6 ±0.2 ***	0.8 ±0.3 ^ns^	0.3 ±0.1 ^ns^	8.8 ±1.0 ^ns^
1.0 mg/kg RP-101075	0.8 ±0.2 ^ns^	1.7 ±0.2 ^ns^	1.4 ±0.2 ^ns^	0.3 ±0.2 ^ns^	1.1 ±0.2 ****	0.4 ±0.2 ^ns^	0.1 ±0.1 *	5.6 ±1.0 **
3.0 mg/kg RP-101075	0.8 ±0.2 ^ns^	1.9 ±0.2 ^ns^	1.5 ±0.2 ^ns^	0.1 ±0.1 *	1.0 ±0.1 ****	0.2 ±0.1 **	0.1 ±0.1 *	5.5 ±0.7 **
Cyclophosphamide	0.4 ±0.1 **	1.2 ±0.1 ***	1.0 ±0.2 ***	0.1 ±0.1 *	0.3 ±0.1 ****	0.1 ±0.1 **	0 ±0 **	3.0 ±0.5 ****

NA, not applicable; SEM, standard error of the mean.

Note: scores for animals that died on-study were censored.

*p <0.05

**p <0.01

***p <0.001

****p <0.0001

ns = not significant, for treatment versus vehicle-treated animals.

Specifically, RPC1063 treatment resulted in a dose-dependent decrease in multiple aspects of the severity of glomerular lesions, including mesangial expansion, endocapillary proliferation, and glomerular deposits, compared with the vehicle-treated control group. No significant changes were noted in extracapillary proliferation (Table 4). As shown in Fig 4A, RPC1063 significantly reduced the combined score for glomerular lesions. In contrast, RP-101075 demonstrated a trend but was not significantly different (Table 5 and Fig 4B). Treatment with RPC1063 (Table 4) and RP-101075 (Table 5) both resulted in a significant dose-dependent decrease in the severity scores for tubular atrophy, interstitial infiltrates, and interstitial fibrosis. The combined score for tubular and interstitial lesions was significantly reduced in a dose-dependent manner for both RPC1063 and RP-101075 (Fig 4C and 4D). The degree of inflammatory infiltrates observed in a vehicle-treated animal compared with reduced inflammatory infiltrates observed in a mouse treated with 3 mg/kg RPC1063 is shown in Fig 4E and 4F.

### RP-101075 does not significantly reduce IgG deposition in kidney or anti-dsDNA antibody production

**Fig 5:**
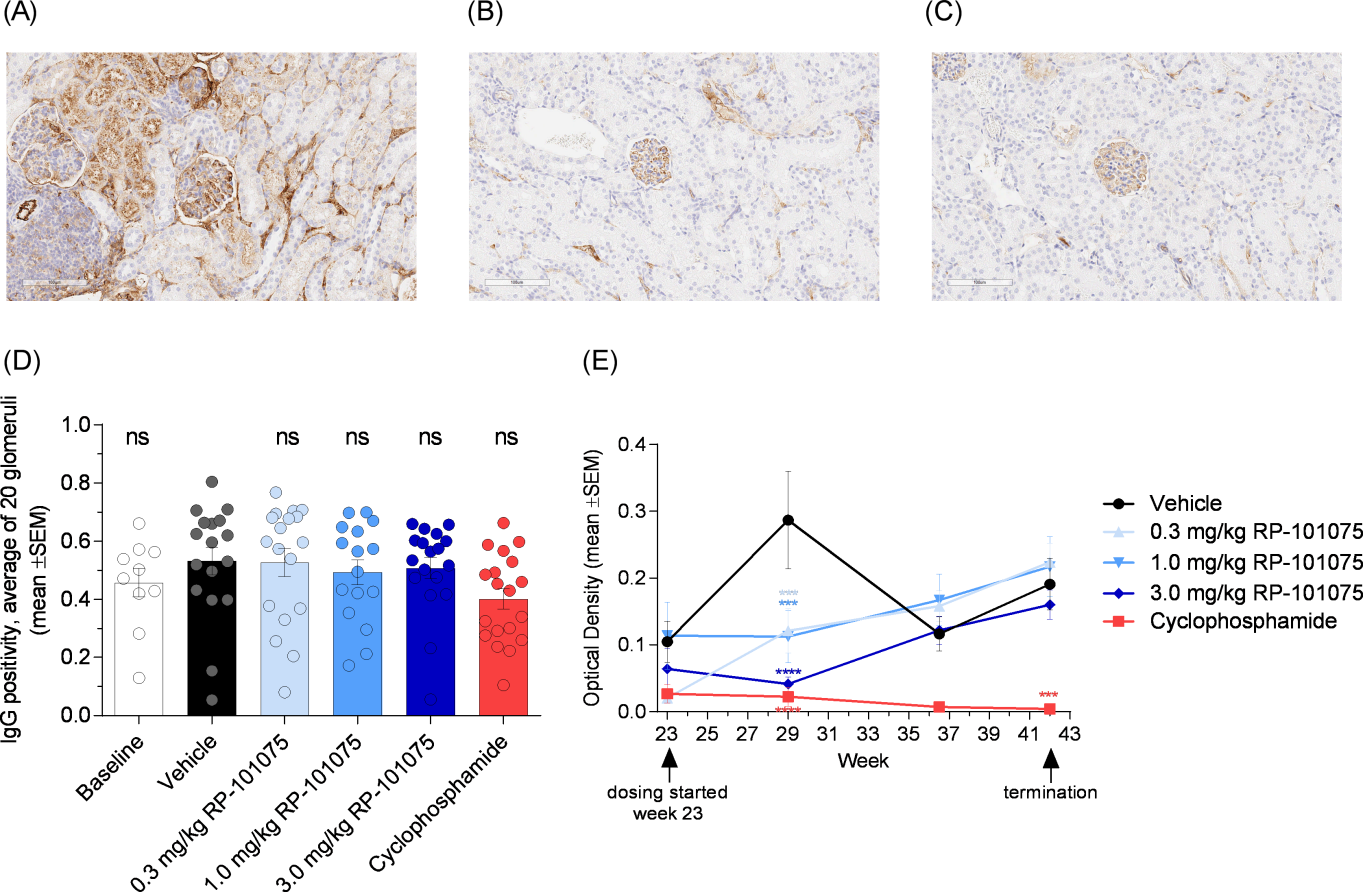
IgG deposition and anti-dsDNA antibody titers. Glomeruli stained for IgG for (A) vehicle, (B) 3.0 mg/kg RP 101075 and (C) cyclophosphamide treated mice. (D) Quantitation of IgG positivity, measured as the average of 20 glomeruli per kidney (mean ± SEM). (E) Anti dsDNA antibody titers as determined by ELISA (presented as mean ± SEM). Baseline data collected from NZBWF1 mice sacrificed at week 23 prior to dosing initiation. *p <0.05, **p <0.01, ***p <0.001, ****p <0.0001, ns = not significant, for treatment versus vehicle-treated animals.

As shown in Fig 5A–5C, individual glomeruli within the kidney showed slightly less IgG staining following treatment with RP-101075 and cyclophosphamide. However, when quantified for IgG positivity, there was no significant difference in IgG deposition comparing mice treated with vehicle to either RP-101075 or cyclophosphamide treatment (Fig 5D). Furthermore, no significant reductions were observed in complement C3 staining following RP-101075 treatment (Figure C in S1 File). Ando *et al*. observed similar and consistent changes in IgG and C3 deposition in mesangial regions following FTY720 treatment in BXSB mice, but again no significant difference when the IgG and C3 deposition was evaluated by relative fluorescence signal intensity per pixel [19].

In addition, antibody titers were measured at weeks 23, 29, 36, and 42. A transient drop in anti-dsDNA antibody titers was observed at week 29 with RP-101075 treatment, however, this was not maintained for the duration of the study (Fig 5E). This is consistent with a previous publication that showed no significant change in anti-dsDNA antibody titers in NZBWF1 mice treated with FTY720 [18].

### RP-101075 treatment results in a unique gene expression signature in NZBWF1 mice

**Fig 6:**
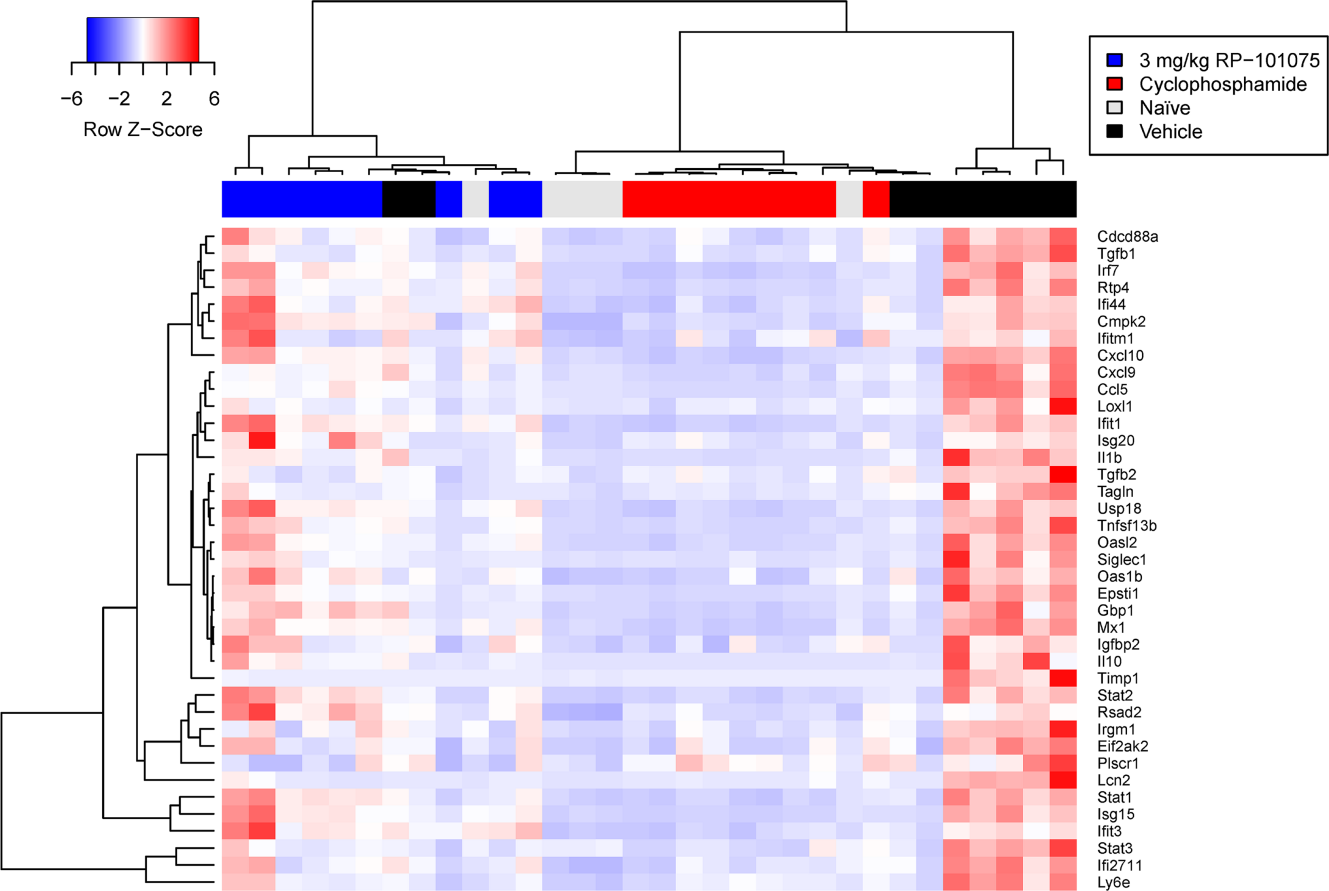
Heatmap of samples clustered by global gene expression, showing samples generally cluster by treatment group. The heatmap was generated by performing unsupervised hierarchical clustering of the gene expression data, and represents the natural grouping of patients according to the similarity of their gene expression profiles, as represented in the dendrogram. This clustering is based only on the gene expression data, and is done without respect to the treatment group. The dendrogram arm lengths are a measure of sample similarity, and they provide a simplified view of which samples are most similar to one another.

To identify gene expression changes that occur within the kidneys, and the impact of RP-101075 treatment, an mRNA panel focused on fibrotic, inflammation, and IFN-induced genes was analyzed. A heatmap of gene expression for all measured genes, along with a clustering dendrogram derived from global expression is shown in Fig 6. Clustering was performed in an unsupervised manner by application of Ward’s method of agglomerative hierarchical clustering, using Euclidean distances between samples. The dendrogram clusters show enrichment of individual treatment groups, indicating distinct transcriptional profiles for each group. Of note, the RP-101075-enriched cluster on the left, the cyclophosphamide-enriched cluster in the center, and the vehicle-enriched cluster on the right.

**Fig 7:**
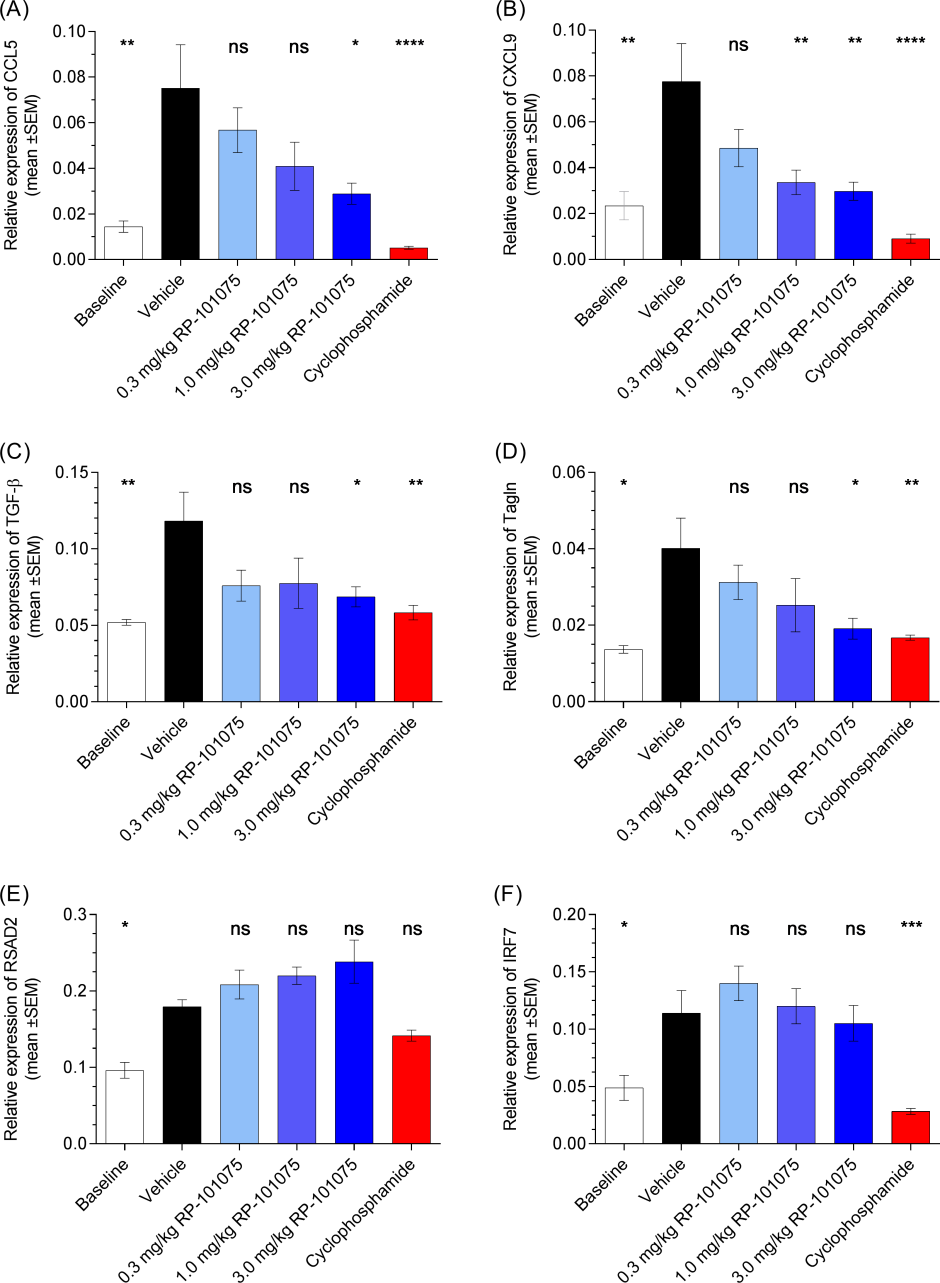
Relative mRNA gene expression in kidneys isolated from NZBWF1 mice. (A, B) inflammatory genes CCL5 and CXCL9, (C, D) pro-fibrotic genes TGF-β and TAGLN, and (E, F) IFN-regulated genes RSAD2 and IRF7. Data presented as mean ± SEM. Baseline data collected from NZBWF1 mice sacrificed at week 23 prior to dosing initiation. *p <0.05, **p <0.01, ***p <0.001, ****p <0.0001, ns = not significant, for treatment versus vehicle-treated animals.

The left-most cluster containing all RP-101075 samples is highly distinct from the other clusters, as evidenced by the fact that the first bifurcation of the dendrogram separates these samples from the other treatment groups. The center cluster of the cyclophosphamide samples is also highly distinct and connected by short dendrogram arms, indicating high transcriptional similarity between samples in this treatment group. These data suggest that although both RP-101075 and cyclophosphamide are efficacious in the SLE model at improving kidney function, they do so by transcriptionally distinct mechanisms. Overall, RP-101075 suppressed expression of profibrotic (e.g., TGF-β, TAGLN) and inflammatory genes (e.g., CCL5, CXCL9) but was less effective at impacting IFN-inducible genes (e.g., RSAD2, IRF7) (Fig 7 and Figure D in S1 File).

### Lymphocytes and pDC are reduced in the spleens of mice treated with RP-101075

NZBWF1 mice treated with RP-101075 had a general dose-dependent reduction in lymphocytes in the spleen following 20 weeks of treatment (Table 6). As expected, a reduction in naïve CD4^+^ T cells was observed as these cells are preferentially retained in the lymph nodes due to agonist-induced internalization of S1P_R1_ by RP-101075 and a consequent inability to exit. In addition, the reduction in lymphocytes in the spleen included several antibody-producing B cell populations and activated CD4^+^ T cell populations. Furthermore, a reduction in pDC, the primary IFNα-producing immune cell in SLE, was also observed in RP-101075 treated NZBWF1 mice.

**Table 6:** Spleen immunophenotyping.

Cell type	Mean percent reduction versus vehicle in cells treated with:			
	Cyclophosphamide	0.3 mg/kg RP-101075	1.0 mg/kg RP-101075	3.0 mg/kg RP-101075
Total splenocytes	81 ****	46 **	64 ****	63 ****
CD19^+^ B cells	92 ****	44 **	69 ****	67 ****
Marginal zone B cells	75 ****	42 *	62 ***	60 ***
Marginal zone progenitor B cells	82 ***	34 s^ns^	67 **	65 **
Germinal center B cells	99 ****	15 ^ns^	48 ^ns^	51 *
Follicular B cells	96 ****	59 ***	81 ****	79 ****
Plasma cells	18 ^ns^	32 ^ns^	41 *	30 ^ns^
CD4^+^ T cells	84 ****	53 **	74 ****	75 ****
CD8^+^ T cells	46 ****	48 ****	69 ****	62 ****
Activated CD4^+^ T cells	93 ****	50 *	72 ***	75 ***
Naïve CD4^+^ T cells	21 ^ns^	84 ****	89 ****	81 ****
pDC	47 **	34 *	64 ****	62 ****

pDC, plasmacytoid dendritic cells.

*p <0.05

**p <0.01

***p <0.001

****p <0.0001

ns = not significant, for treatment versus vehicle-treated animals.

### IFNAR1 expression on pDC is not significantly reduced following *in vivo* RP-101075 treatment

**Fig 8:**
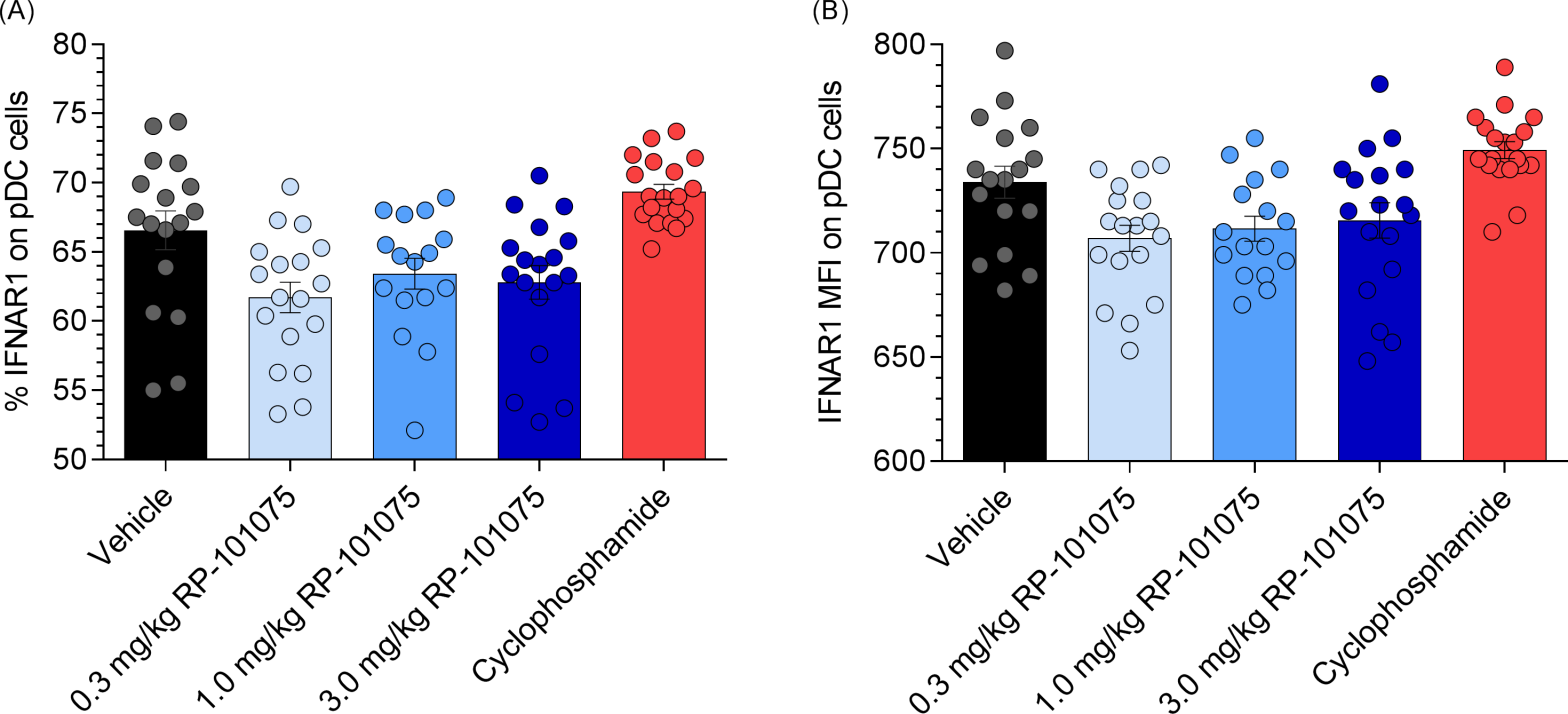
IFNAR1 expression on pDC isolated from NZBWF1 spleens. (A) Percentage of pDCs expressing IFNAR1. (B) IFNAR1 mean fluorescence intensity (MFI) on pDC as determined by flow cytometry.

Teijaro *et al*. [21] demonstrated *in vitro* that an S1P_R1_ agonist (CYM-5442) promoted IFNAR1 degradation in cultured pDCs. We extended this hypothesis by analyzing *in vivo* IFNAR1 expression on splenic pDCs isolated from NZBWF1 mice, following long-term treatment with RP-101075. Following 20 weeks of RP-101075 treatment, there was a reduction in the percentage of pDCs expressing IFNAR1 versus vehicle (Fig 8A), however it did not achieve statistical significance. Of those pDCs still expressing IFNAR1 following RP-101075 treatment, there was also less IFNAR1 on the cell surface, but again, this did not reach statistical significance (Fig 8B).

## Discussion

The NZBWF1 mouse model has several characteristics of human SLE including immune-complex glomerulonephritis, loss of kidney function, and autoantibody production [26]. Ozanimod (RPC1063) and its metabolite RP-101075, both selective S1P_R1_ and S1P_R5_ modulators, demonstrated significant therapeutic benefit in this murine SLE model. Mice treated with ozanimod and RP-101075 showed a dose-dependent reduction in proteinuria and serum BUN, and improvements in multiple parameters of kidney pathology, including reduced interstitial infiltrates, tubular atrophy, and interstitial fibrosis. Furthermore, ozanimod demonstrated a reduction in multiple components of glomerular lesions, including reduced mesangial expansion, endocapillary proliferation and glomerular deposits. The reduction in immune cell infiltration into the kidney is of particular clinical significance as ectopic lymphoid structures are a hallmark of many autoimmune diseases, including SLE [27–30]. Together, this data demonstrates that ozanimod and RP-101075 reduce kidney inflammation and improve kidney function.

Modulation of S1P_R1_ reduces circulating T and B lymphocytes through retention in secondary lymphoid tissues. In SLE, B cells have garnered significant interest as drug targets to reduce autoantibody production. Belimumab, an anti-BAFF/BLyS antibody was approved for SLE in 2011 and rituximab is used in patients with refractory disease, despite negative results from clinical trials [31]. Of particular interest from this NZBWF1 study, both ozanimod and RP-101075 significantly reduce peripheral and splenic B cell subsets which may contribute to the reduction in disease activity. However, unlike cyclophosphamide and rituximab, which kill B cells directly [32–34], ozanimod and RP-101075 do not. What remains to be investigated is whether sequestration of both B and T cells in SLE patients by an S1P_R1_-specific modulator can drive the same therapeutic benefit, as opposed to killing B cells directly, and whether engaging S1P_R5_ contributes to clinical benefit.

Despite a reduction in circulating T and B lymphocytes, RP-101075 did not have a sustained effect on circulating anti-dsDNA antibodies. This result is consistent with what was observed with FTY720, a pan-S1P_R1,R3,R4,R5_ modulator, in the NZBWF1 model [18]. Vaccination in MS patients treated with FTY720 (fingolimod) demonstrates that these patients can produce vaccine-specific antibodies, however, the magnitude of response is reduced and the avidity of IgG-specific antibodies does not increase [35]. Therefore, although the anti-dsDNA antibody response in the NZBWF1 mice treated with RP-101075 does not change, future studies investigating specific antibody avidity may be informative. In addition, neither RP-101075 nor cyclophosphamide significantly reduced IgG nor complement deposition in the glomerulus. Despite this, both intervention strategies significantly improved kidney function suggesting a disconnect between the IgG and complement deposition with loss of kidney function in this SLE model. It is currently unclear whether an S1P_R1,R5_ modulator can produce a sustained reduction of autoantibodies in SLE patients and whether this reduction is require to achieve therapeutic benefits on kidney function.

To further explore the potential gene expression changes induced by S1P_R1/R5_ modulation *in vivo*, the expression of inflammatory, fibrotic and IFNα related genes were analyzed in the kidney of mice treated with RP-101075. Cluster and dendogram data analysis revealed that cyclophosphamide and RP-101075 treatment groups fall into distinct and separate self-organizing clusters (Fig 6). RP-101075 dramatically suppressed expression of genes encoding pro-fibrotic proteins such as TGF-β, Transgelin, lipocalin, TIMP1, and LOXL1, and expression of genes encoding pro-inflammatory proteins such as IL-1β, CCL5, and CXCL9. Whereas cyclophosphamide suppressed expression of the majority of the IFN Type I inducible genes, RP-101075 had a less pronounced effect and onlySTAT3, Mx1, Ly6E, EPSTI1, OAS1β, Siglec1, and IFI27L1 were suppressed in a dose-dependent and numerical manner, yet they did not achieve statistical significance.

The IFNα pathway has been identified as a potential therapeutic target in SLE and encouraging data has emerged from clinical trials. Sifalimumab (MedImmune LLC) and rontalizumab (Genentech) are monoclonal antibodies that target IFNα, while anifrolumab (MedImmune LLC) targets the IFNα receptor. Patients with a high IFN gene signature were more responsive to anifrolumab in a phase II clinical trial [36]. One might hypothesize that an S1P_R1_,_R5_ modulator may provide therapeutic benefit to specific SLE patient populations with distinct IFN profiles. Recently, it was proposed that S1P_R1_ expression on pDCs modulates IFNα receptor. In a series of *in vitro* experiments, a selective S1P_R1_ agonist, CYM-5442, was able to drive IFNAR1 degradation, limiting pDC IFNα auto-amplification [21]. Although we did not observe statistically significant changes in the amount of IFNAR1 on the cell surface of pDCs from mice treated with RP-101075, we did see a numerical trend toward reduced IFNAR1 expression. Interestingly, treatment with RP-101075 significantly reduced the number of pDCs in the spleen. Despite the marginal effect of RP-101075 on IFNRA1, it remains possible that reduction of pDCs by RP-101075 may contribute to its therapeutic benefit in the NZBWF1 model. The mechanism of action driving the reduction on pDC numbers is unclear and further studies are needed to determine how these dual S1P_R1,R5_ modulators regulate pDC function during experimental SLE.

Overall, this study demonstrates that both ozanimod and its metabolite RP-101075 are efficacious in the NZBWF1 SLE mouse model. These data emphasize the importance of the S1P_R1,R5_ pathway in the development of disease in this model and highlight S1P_R1_ modulation as a novel and exciting mechanism for the treatment of patients with SLE.

## Supporting information

S1 File **(Figure A) Kidney weights.** Left (A) kidney weights in RPC1063-treated mice at week 42; and left (B) and right (C) kidney weights in RP-101075–treated mice at week 42. *p <0.05, **p <0.01, ***p <0.001, ****p <0.0001, ns = not significant, for treatment versus vehicle-treated animals. **(Figure B) Effect of RP-101075 Treatment on Circulating Lymphocyte Subsets.** (A) CD4^+^ T cells, (B) CD8^+^ T cells, and (C) CD19^+^ B cell counts at weeks 23, 29, and 36.5. **(Figure C) Complement Component C3.** Glomeruli stained for complement component C3: (A) vehicle, (B) 3.0 mg/kg RP-101075, (C) cyclophosphamide. **(Figure D) Relative mRNA Gene Expression.** Inflammatory, pro-fibrotic, and IFN-regulated genes. *p <0.05, **p <0.01, ***p <0.001, ****p <0.0001, ns = not significant, for treatment versus vehicle-treated animals.
(PDF)
