# Supplementary material for: Ozanimod (RPC1063), a selective S1PR1 and S1PR5 modulator, reduces chronic inflammation and alleviates kidney pathology in murine systemic lupus erythematosus
Source: PLoS One. 2018 Apr 2;13(4):e0193236. doi: 10.1371/journal.pone.0193236 (PMC5880347; doi:10.1371/journal.pone.0193236)
Supplement: S1 File — (Figure A) Kidney weights. Left (A) kidney weights in RPC1063-treated mice at week 42; and left (B) and right (C) kidney weights in RP-101075–treated mice at week 42. *p <0.05, **p <0.01, ***p <0.001, ****p <0.0001, ns = not significant, for treatment versus vehicle-treated animals. (Figure B) Effect of RP-101075 Treatment on Circulating Lymphocyte Subsets. (A) CD4+ T cells, (B) CD8+ T cells, and (C) CD19+ B cell counts at weeks 23, 29, and 36.5. (Figure C) Complement Component C3. Glomeruli stained for complement component C3: (A) vehicle, (B) 3.0 mg/kg RP-101075, (C) cyclophosphamide. (Figure D) Relative mRNA Gene Expression. Inflammatory, pro-fibrotic, and IFN-regulated genes. *p <0.05, **p <0.01, ***p <0.001, ****p <0.0001, ns = not significant, for treatment versus vehicle-treated animals. (PDF) [file pone.0193236.s001.pdf]

SUPPLEMENTAL INFORMATION

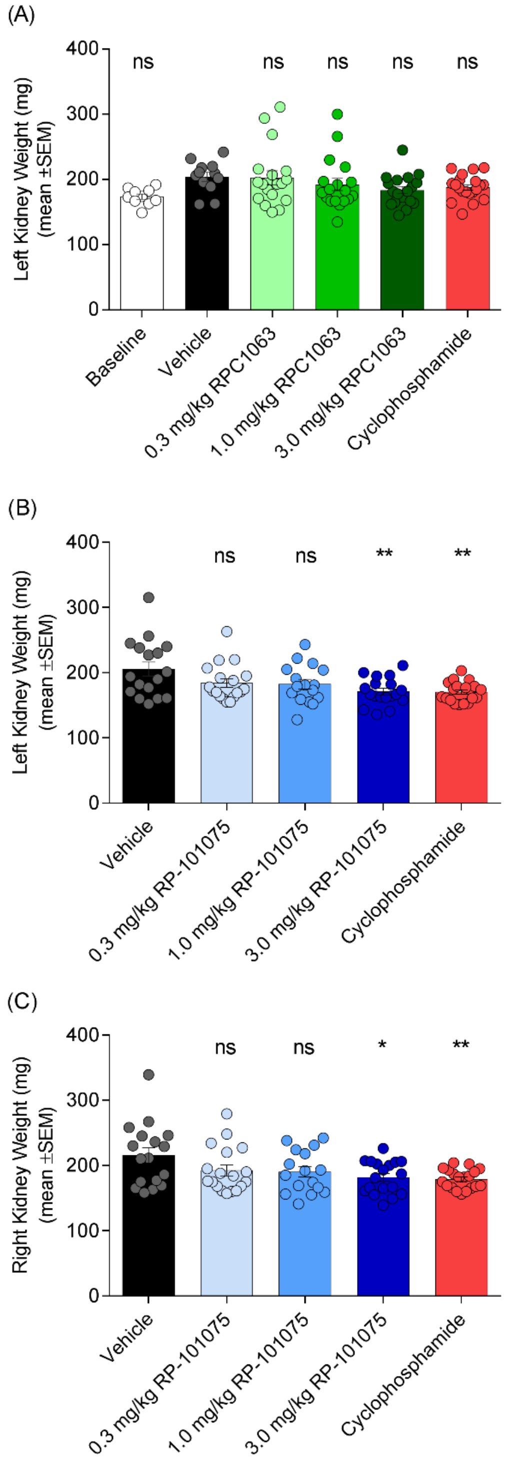

(Figure A) Kidney weights. Left (A) kidney weights in RPC1063-treated mice at week 42; and left (B) and right (C) kidney weights in RP-101075–treated mice at week 42. **p* <0.05, ***p* <0.01, ****p* <0.001, *****p* <0.0001, ns = not significant, for treatment versus vehicle-treated animals.

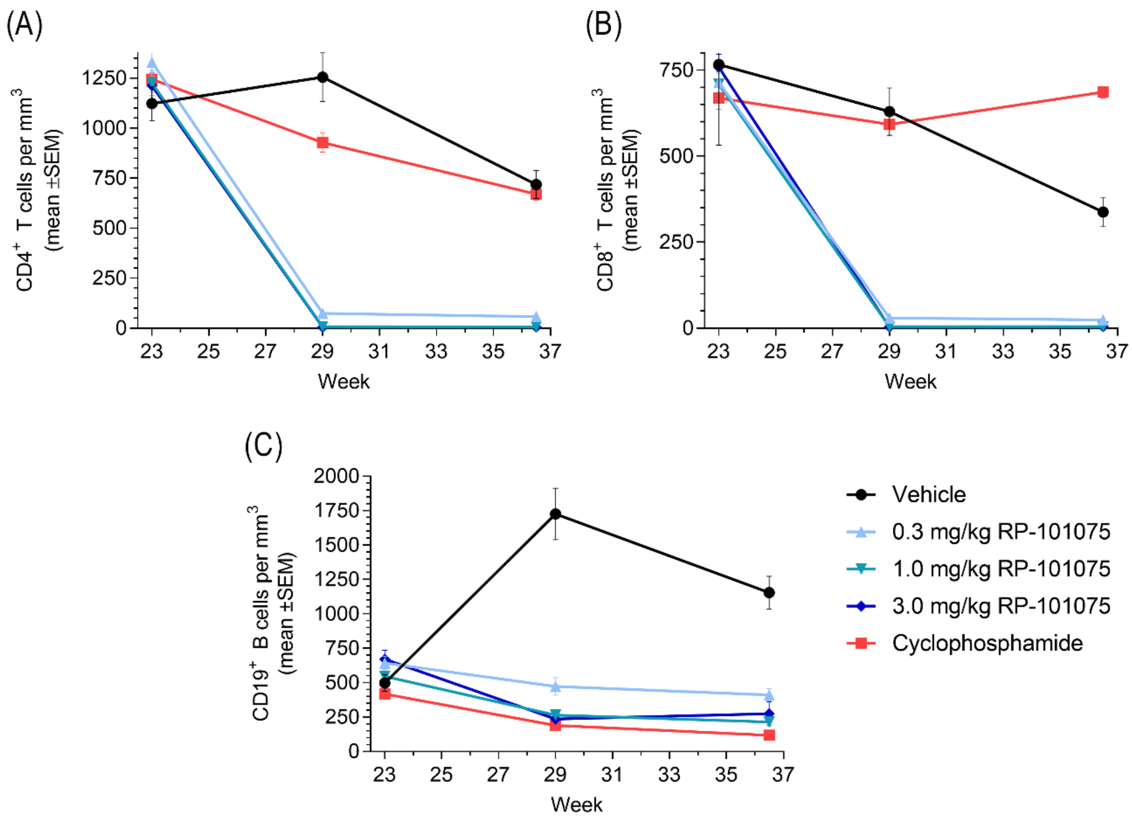

(Figure B) Effect of RP-101075 Treatment on Circulating Lymphocyte Subsets. (A) $CD4^+$ T cells, (B) $CD8^+$ T cells, and (C) $CD19^+$ B cell counts at weeks 23, 29, and 36.5.

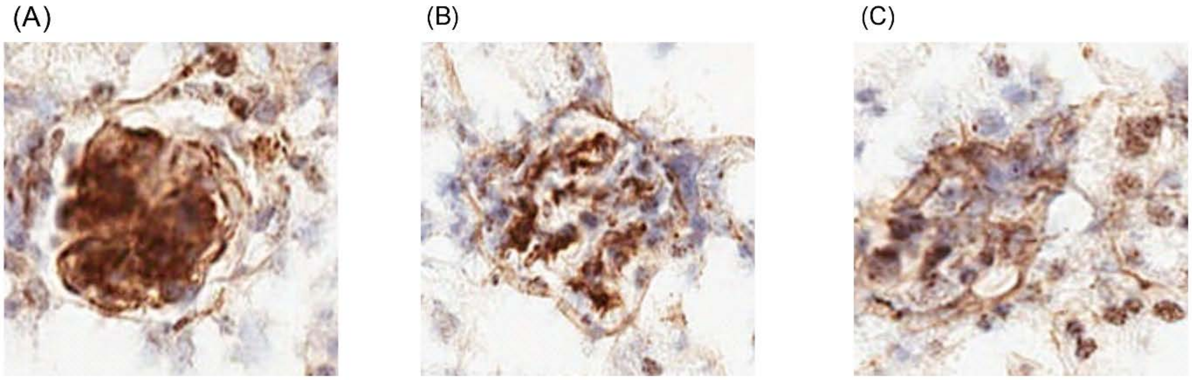

(Figure C) Complement Component C3. Glomeruli stained for complement component C3: (A) vehicle, (B) 3.0 mg/kg RP-101075, (C) cyclophosphamide.

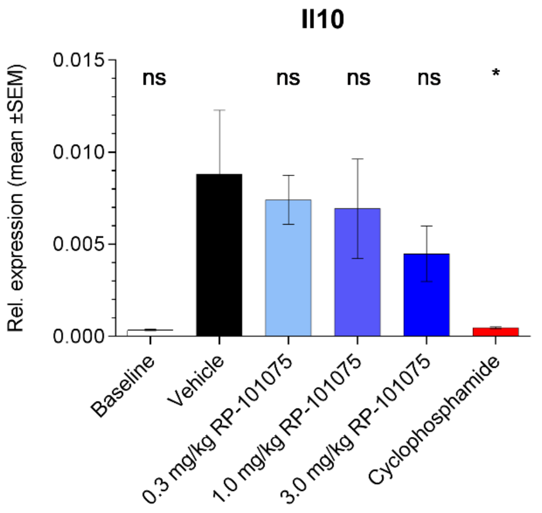
Il10
Rel. expression (mean ±SEM)
0.015
0.010
0.005
0.000
ns
ns
ns
ns
*
Baseline
Vehicle
0.3 mg/kg RP-101075
1.0 mg/kg RP-101075
3.0 mg/kg RP-101075
Cyclophosphamide

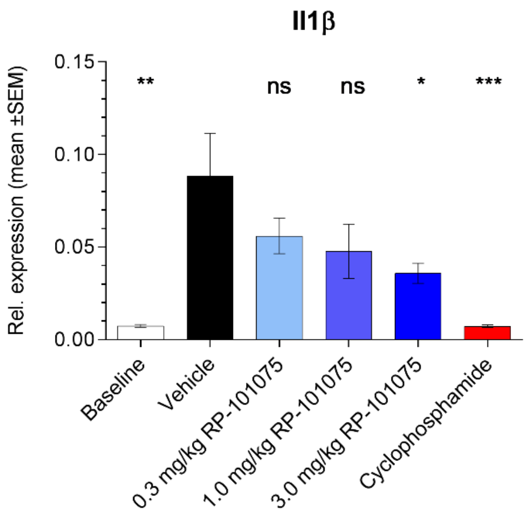
Il1β
Rel. expression (mean ±SEM)
0.15
0.10
0.05
0.00
**
ns
ns
*
***
Baseline
Vehicle
0.3 mg/kg RP-101075
1.0 mg/kg RP-101075
3.0 mg/kg RP-101075
Cyclophosphamide

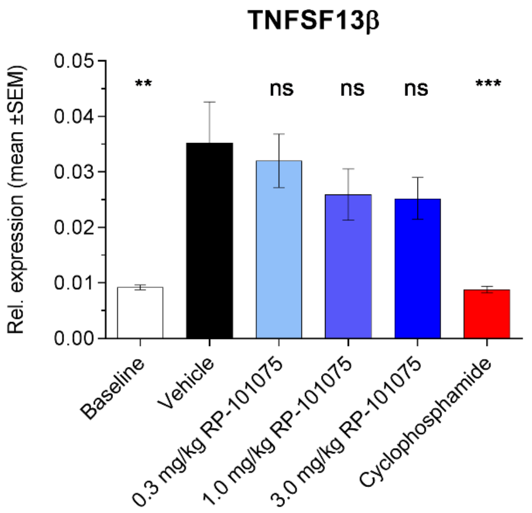
TNFSF13β
Rel. expression (mean ±SEM)
0.05
0.04
0.03
0.02
0.01
0.00
**
ns
ns
ns
***
Baseline
Vehicle
0.3 mg/kg RP-101075
1.0 mg/kg RP-101075
3.0 mg/kg RP-101075
Cyclophosphamide

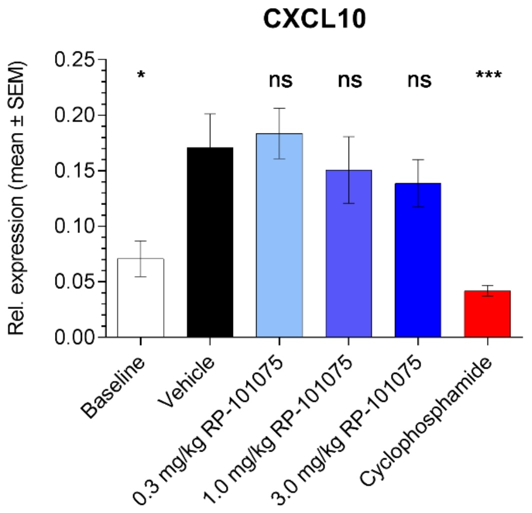
CXCL10
Rel. expression (mean ± SEM)
0.25
0.20
0.15
0.10
0.05
0.00
*
ns
ns
ns
***
Baseline
Vehicle
0.3 mg/kg RP-101075
1.0 mg/kg RP-101075
3.0 mg/kg RP-101075
Cyclophosphamide

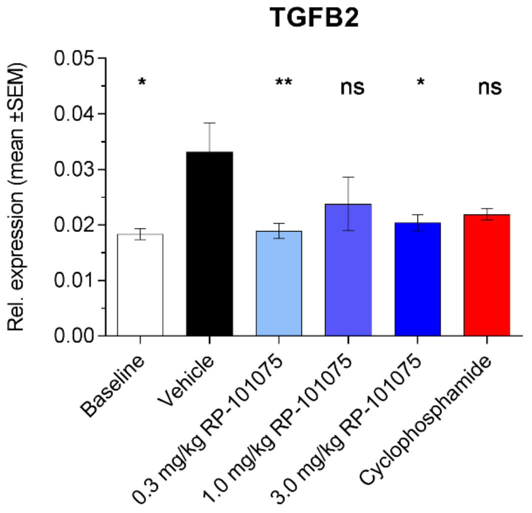
TGFB2
Rel. expression (mean ±SEM)
0.05
0.04
0.03
0.02
0.01
0.00
*
**
ns
*
ns
Baseline
Vehicle
0.3 mg/kg RP-101075
1.0 mg/kg RP-101075
3.0 mg/kg RP-101075
Cyclophosphamide

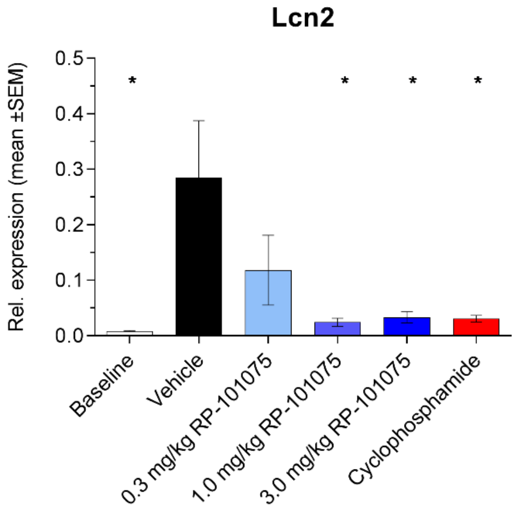
Lcn2
Rel. expression (mean ±SEM)
0.5
0.4
0.3
0.2
0.1
0.0
*
*
*
*
Baseline
Vehicle
0.3 mg/kg RP-101075
1.0 mg/kg RP-101075
3.0 mg/kg RP-101075
Cyclophosphamide

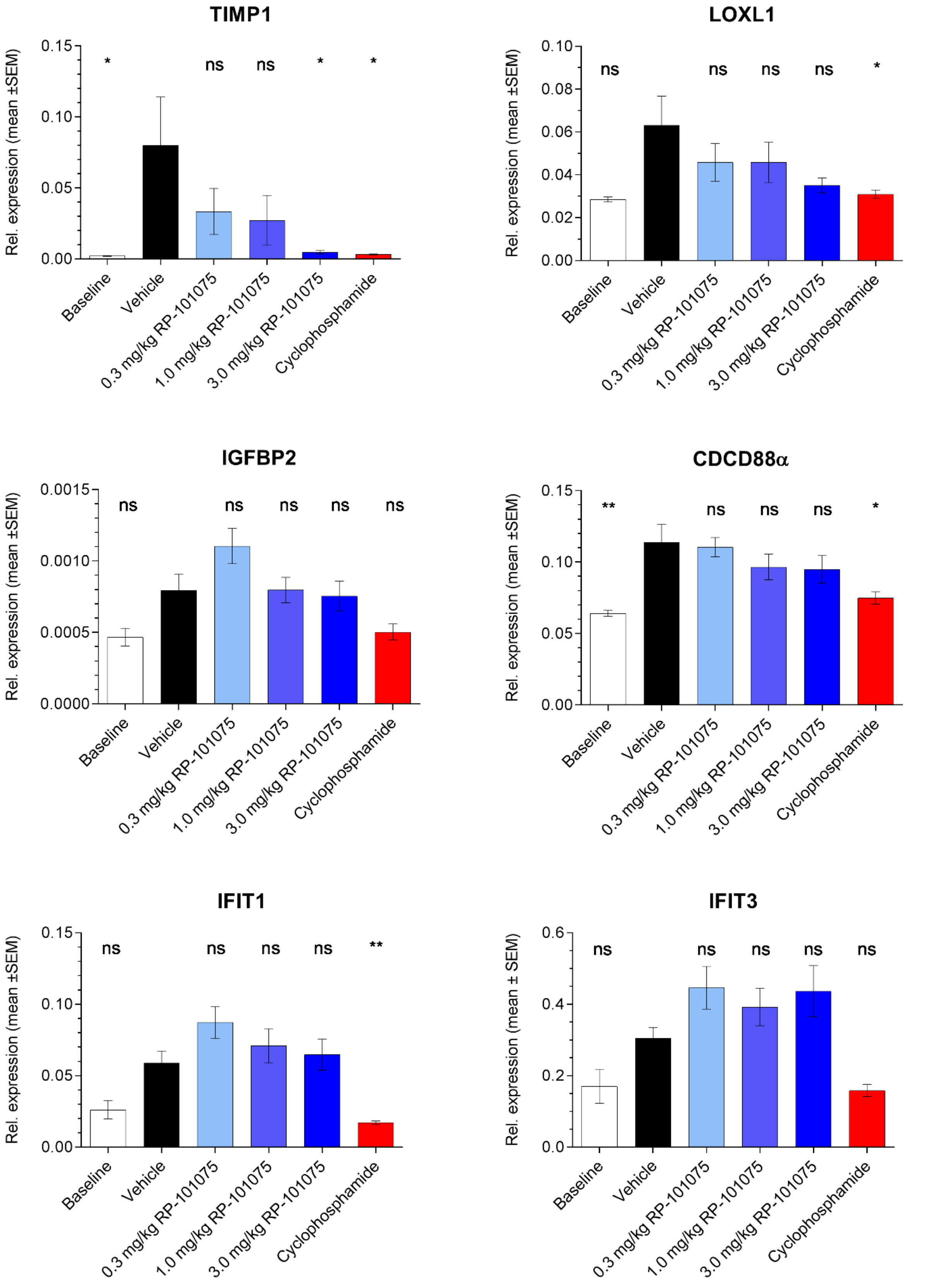
TIMP1
LOXL1
IGFBP2
CDCD88α
IFIT1
IFIT3
Rel. expression (mean ±SEM)
Rel. expression (mean ± SEM)
ns
*
**
Baseline
Vehicle
0.3 mg/kg RP-101075
1.0 mg/kg RP-101075
3.0 mg/kg RP-101075
Cyclophosphamide

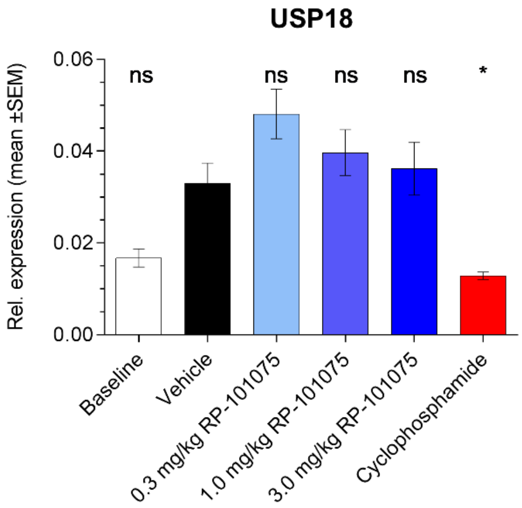
USP18
Rel. expression (mean ±SEM)
0.06
0.04
0.02
0.00
ns
ns
ns
ns
*
Baseline
Vehicle
0.3 mg/kg RP-101075
1.0 mg/kg RP-101075
3.0 mg/kg RP-101075
Cyclophosphamide

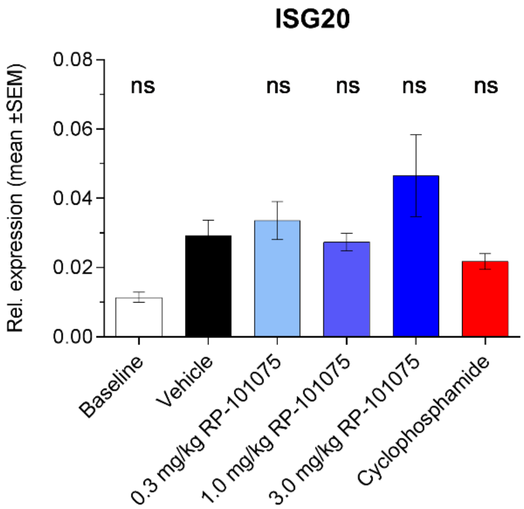
ISG20
Rel. expression (mean ±SEM)
0.08
0.06
0.04
0.02
0.00
ns
ns
ns
ns
ns
Baseline
Vehicle
0.3 mg/kg RP-101075
1.0 mg/kg RP-101075
3.0 mg/kg RP-101075
Cyclophosphamide

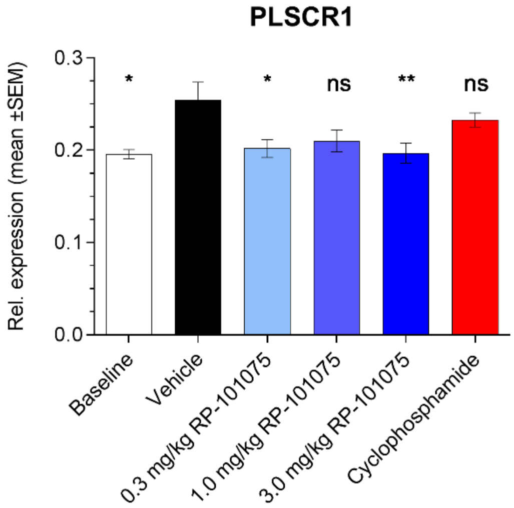
PLSCR1
Rel. expression (mean ±SEM)
0.3
0.2
0.1
0.0
*
*
ns
**
ns
Baseline
Vehicle
0.3 mg/kg RP-101075
1.0 mg/kg RP-101075
3.0 mg/kg RP-101075
Cyclophosphamide

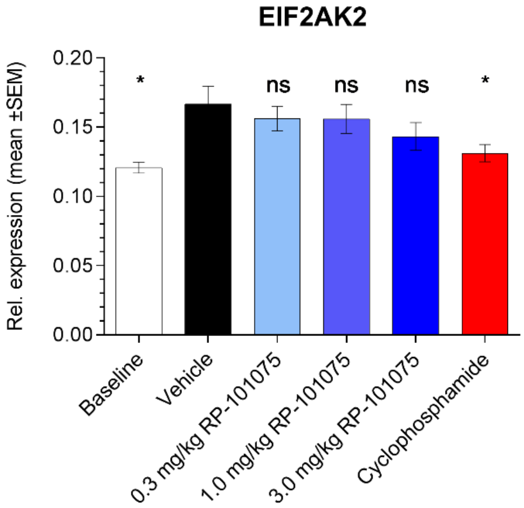
EIF2AK2
Rel. expression (mean ±SEM)
0.20
0.15
0.10
0.05
0.00
*
ns
ns
ns
*
Baseline
Vehicle
0.3 mg/kg RP-101075
1.0 mg/kg RP-101075
3.0 mg/kg RP-101075
Cyclophosphamide

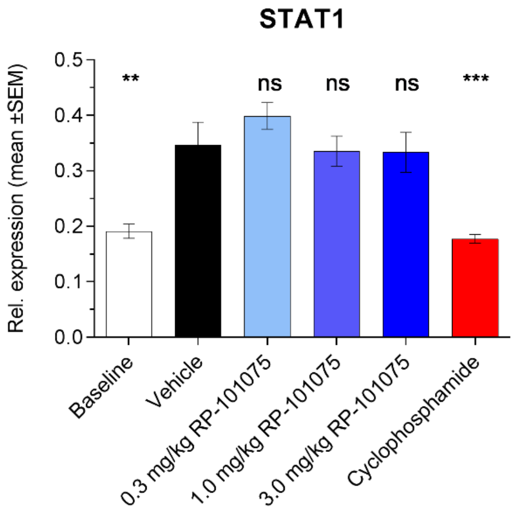
STAT1
Rel. expression (mean ±SEM)
0.5
0.4
0.3
0.2
0.1
0.0
**
ns
ns
ns
***
Baseline
Vehicle
0.3 mg/kg RP-101075
1.0 mg/kg RP-101075
3.0 mg/kg RP-101075
Cyclophosphamide

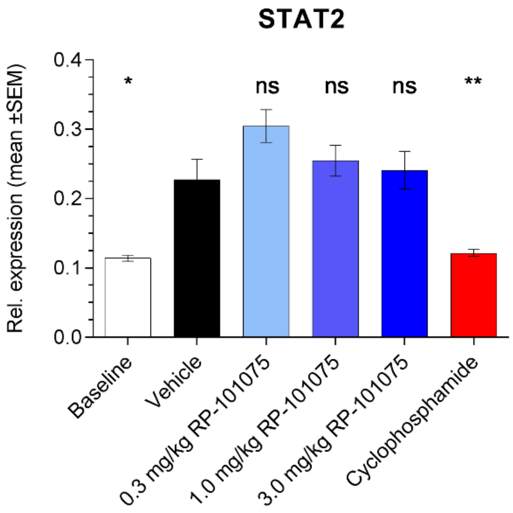
STAT2
Rel. expression (mean ±SEM)
0.4
0.3
0.2
0.1
0.0
*
ns
ns
ns
**
Baseline
Vehicle
0.3 mg/kg RP-101075
1.0 mg/kg RP-101075
3.0 mg/kg RP-101075
Cyclophosphamide

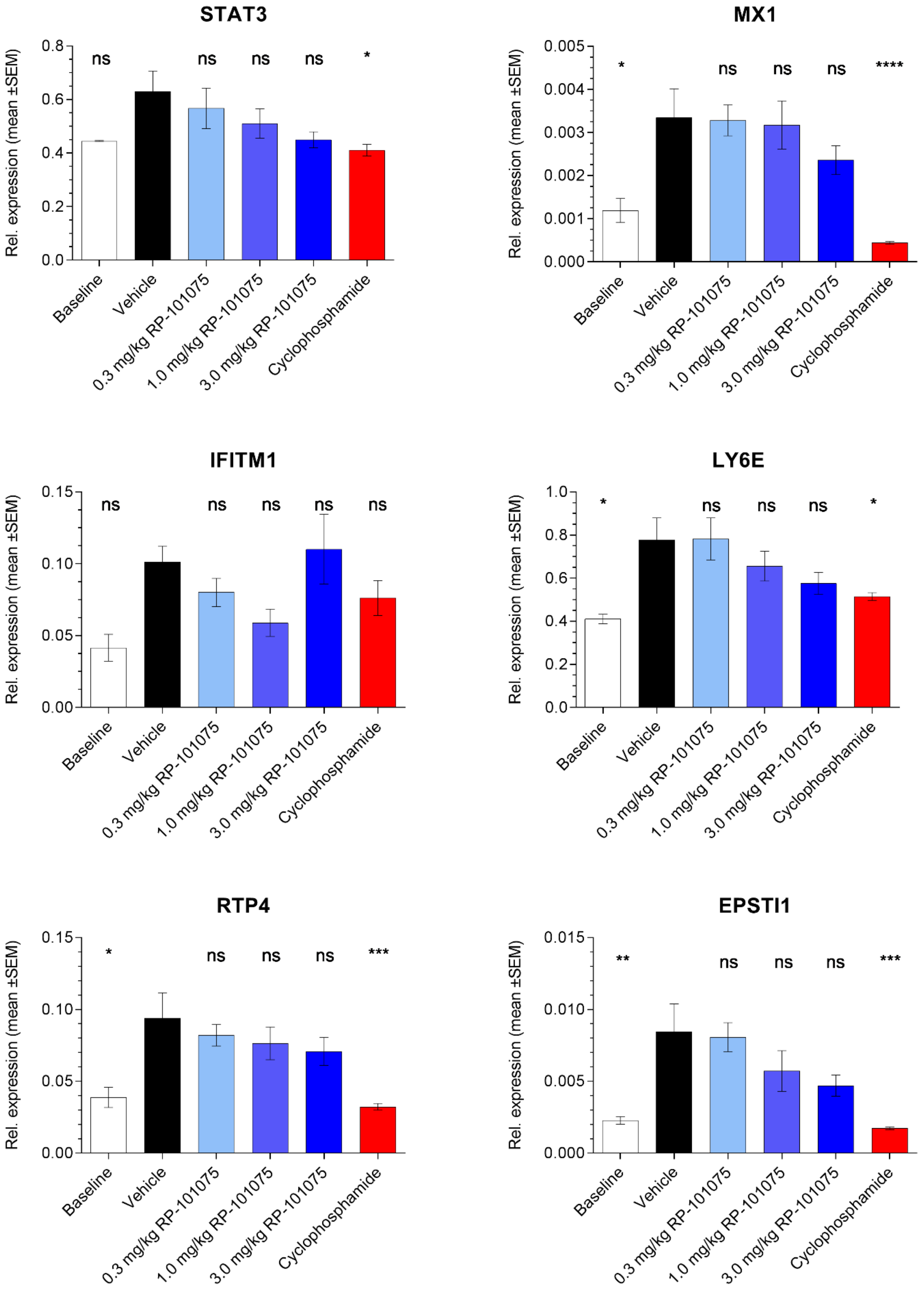
STAT3
Rel. expression (mean ±SEM)
0.0
0.2
0.4
0.6
0.8
ns
ns
ns
ns
*
Baseline
Vehicle
0.3 mg/kg RP-101075
1.0 mg/kg RP-101075
3.0 mg/kg RP-101075
Cyclophosphamide
MX1
Rel. expression (mean ±SEM)
0.000
0.001
0.002
0.003
0.004
0.005
*
ns
ns
ns
****
Baseline
Vehicle
0.3 mg/kg RP-101075
1.0 mg/kg RP-101075
3.0 mg/kg RP-101075
Cyclophosphamide
IFITM1
Rel. expression (mean ±SEM)
0.00
0.05
0.10
0.15
ns
ns
ns
ns
ns
Baseline
Vehicle
0.3 mg/kg RP-101075
1.0 mg/kg RP-101075
3.0 mg/kg RP-101075
Cyclophosphamide
LY6E
Rel. expression (mean ±SEM)
0.0
0.2
0.4
0.6
0.8
1.0
*
ns
ns
ns
*
Baseline
Vehicle
0.3 mg/kg RP-101075
1.0 mg/kg RP-101075
3.0 mg/kg RP-101075
Cyclophosphamide
RTP4
Rel. expression (mean ±SEM)
0.00
0.05
0.10
0.15
*
ns
ns
ns
***
Baseline
Vehicle
0.3 mg/kg RP-101075
1.0 mg/kg RP-101075
3.0 mg/kg RP-101075
Cyclophosphamide
EPSTI1
Rel. expression (mean ±SEM)
0.000
0.005
0.010
0.015
**
ns
ns
ns
***
Baseline
Vehicle
0.3 mg/kg RP-101075
1.0 mg/kg RP-101075
3.0 mg/kg RP-101075
Cyclophosphamide

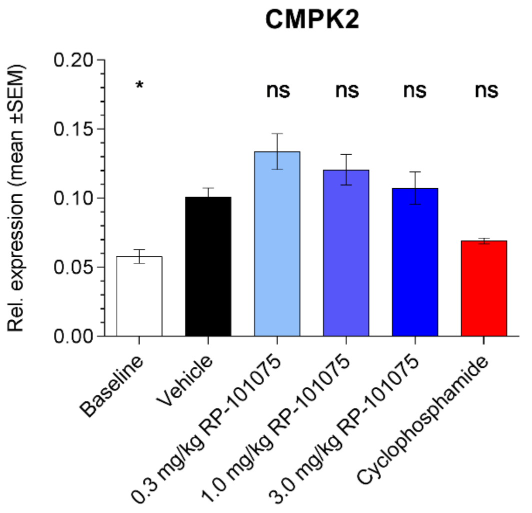
CMPK2
Rel. expression (mean ±SEM)
0.20
0.15
0.10
0.05
0.00
*
ns
ns
ns
ns
Baseline
Vehicle
0.3 mg/kg RP-101075
1.0 mg/kg RP-101075
3.0 mg/kg RP-101075
Cyclophosphamide

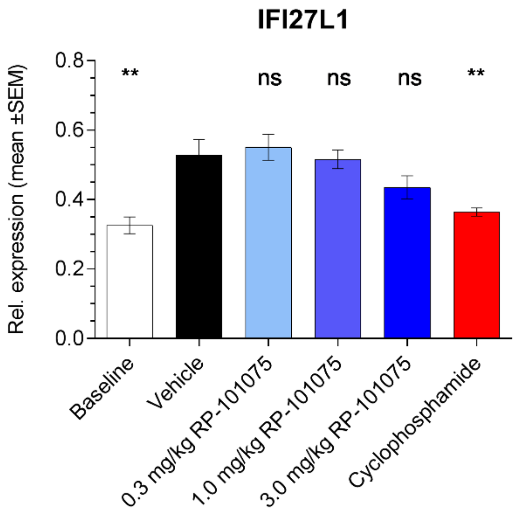
IFI27L1
Rel. expression (mean ±SEM)
0.8
0.6
0.4
0.2
0.0
**
ns
ns
ns
**
Baseline
Vehicle
0.3 mg/kg RP-101075
1.0 mg/kg RP-101075
3.0 mg/kg RP-101075
Cyclophosphamide

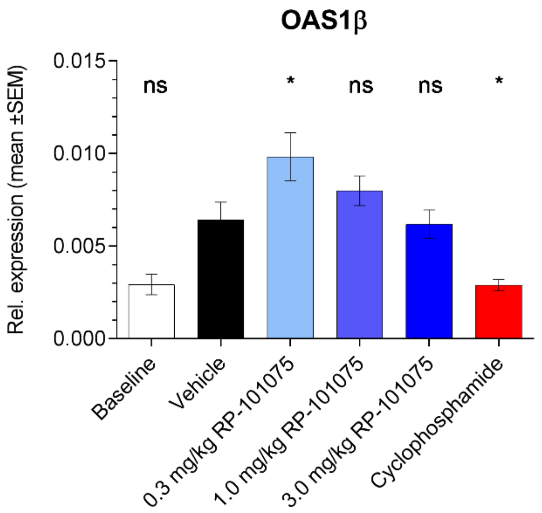
OAS1β
Rel. expression (mean ±SEM)
0.015
0.010
0.005
0.000
ns
*
ns
ns
*
Baseline
Vehicle
0.3 mg/kg RP-101075
1.0 mg/kg RP-101075
3.0 mg/kg RP-101075
Cyclophosphamide

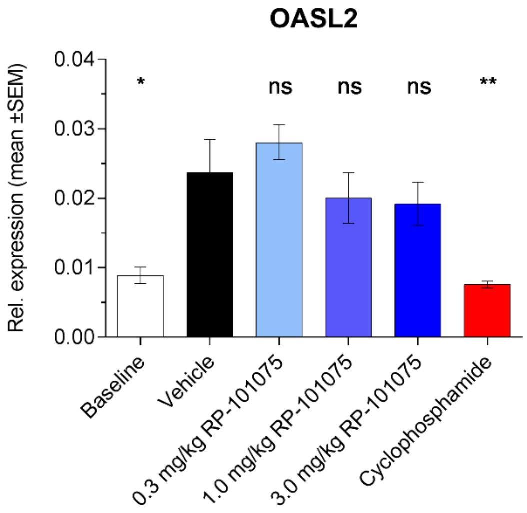
OASL2
Rel. expression (mean ±SEM)
0.04
0.03
0.02
0.01
0.00
*
ns
ns
ns
**
Baseline
Vehicle
0.3 mg/kg RP-101075
1.0 mg/kg RP-101075
3.0 mg/kg RP-101075
Cyclophosphamide

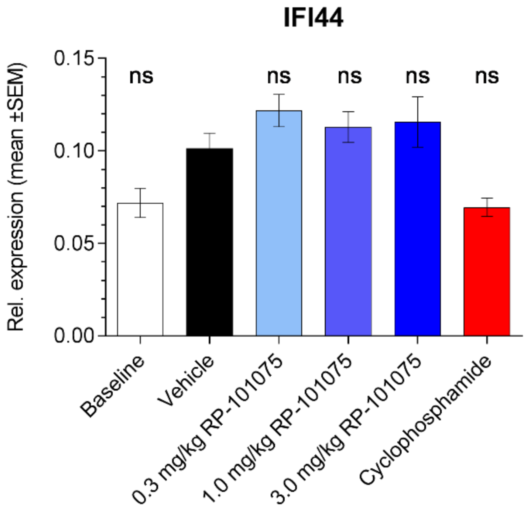
IFI44
Rel. expression (mean ±SEM)
0.15
0.10
0.05
0.00
ns
ns
ns
ns
ns
Baseline
Vehicle
0.3 mg/kg RP-101075
1.0 mg/kg RP-101075
3.0 mg/kg RP-101075
Cyclophosphamide

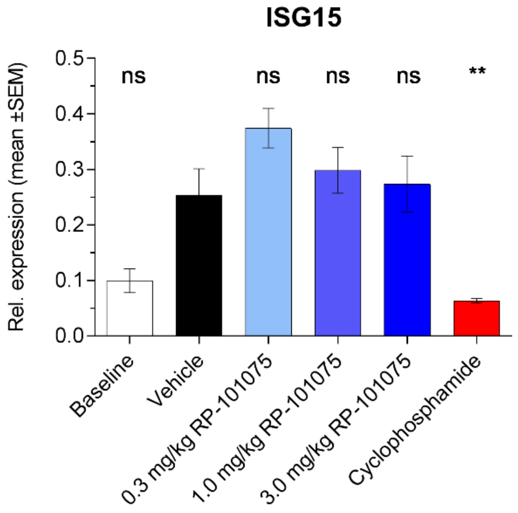
ISG15
Rel. expression (mean ±SEM)
0.5
0.4
0.3
0.2
0.1
0.0
ns
ns
ns
ns
**
Baseline
Vehicle
0.3 mg/kg RP-101075
1.0 mg/kg RP-101075
3.0 mg/kg RP-101075
Cyclophosphamide

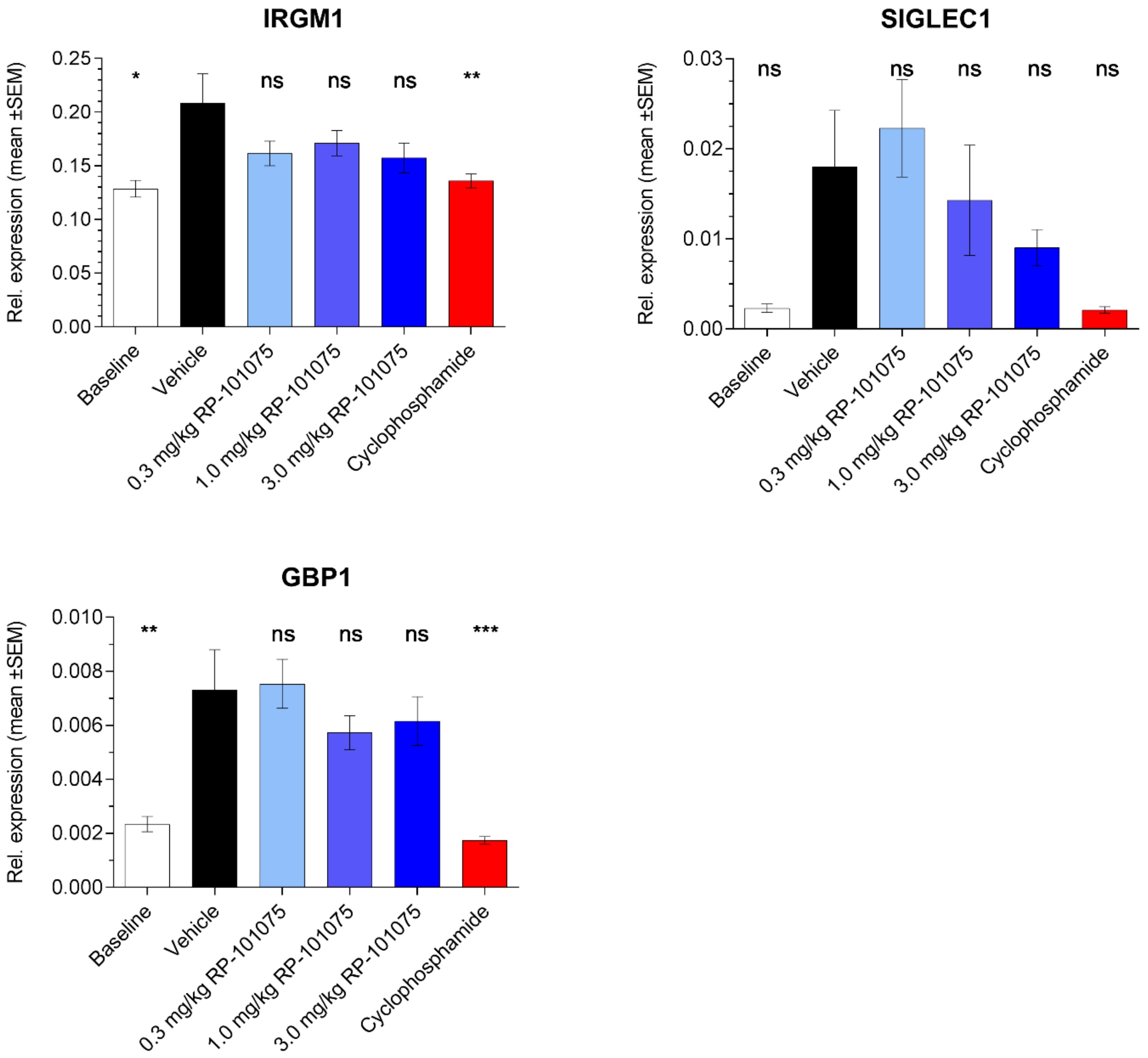

(Figure D) Relative mRNA Gene Expression. Inflammatory, pro-fibrotic, and IFN-regulated genes. *p <0.05, **p <0.01, ***p <0.001, ****p <0.0001, ns = not significant, for treatment versus vehicle-treated animals.
